# On the Importance of Polar Interactions for Complexes Containing Intrinsically Disordered Proteins

**DOI:** 10.1371/journal.pcbi.1003192

**Published:** 2013-08-22

**Authors:** Eric T. C. Wong, Dokyun Na, Jörg Gsponer

**Affiliations:** Centre for High-Throughput Biology, University of British Columbia, East Mall, Vancouver, Canada; Tel Aviv University, Israel

## Abstract

There is a growing recognition for the importance of proteins with large intrinsically disordered (ID) segments in cell signaling and regulation. ID segments in these proteins often harbor regions that mediate molecular recognition. Coupled folding and binding of the recognition regions has been proposed to confer high specificity to interactions involving ID segments. However, researchers recently questioned the origin of the interaction specificity of ID proteins because of the overrepresentation of hydrophobic residues in their interaction interfaces. Here, we focused on the role of polar and charged residues in interactions mediated by ID segments. Making use of the extended nature of most ID segments when in complex with globular proteins, we first identified large numbers of complexes between globular proteins and ID segments by using radius-of-gyration-based selection criteria. Consistent with previous studies, we found the interfaces of these complexes to be enriched in hydrophobic residues, and that these residues contribute significantly to the stability of the interaction interface. However, our analyses also show that polar interactions play a larger role in these complexes than in structured protein complexes. Computational alanine scanning and salt-bridge analysis indicate that interfaces in ID complexes are highly complementary with respect to electrostatics, more so than interfaces of globular proteins. Follow-up calculations of the electrostatic contributions to the free energy of binding uncovered significantly stronger Coulombic interactions in complexes harbouring ID segments than in structured protein complexes. However, they are counter-balanced by even higher polar-desolvation penalties. We propose that polar interactions are a key contributing factor to the observed high specificity of ID segment-mediated interactions.

## Author Summary

Protein-protein interactions are essential to communication and signal integration in cells. For these processes to be precise, interactions between proteins have to be specific and well coordinated. In order to understand the specificity in protein interactions, researches have focused on interfaces between two or more folded proteins. It has been shown that specificity in interactions between folded proteins relies on shape complementarity, hydrogen bonding, and salt-bridge formation. However, many proteins lack a unique folded structure; the so-called intrinsically disordered proteins. These proteins fluctuate between different conformations in isolation but often adopt a single structure when interacting with partner proteins. As many intrinsically disordered proteins are involved in signaling and regulation, their interactions have to be highly specific. The finding that the interaction interfaces of intrinsically disordered proteins are enriched in hydrophobic residues has led to questions regarding the specificity of interactions mediated by this group of proteins. Here, we show that polar and charged residues play a larger role in interfaces that involve intrinsically disordered proteins compared to interfaces that involve only folded proteins. Our results suggest that polar interactions are key contributors to the specificity of interactions that involve intrinsically disordered proteins.

## Introduction

In cells, communication is established principally by protein-protein interactions [1]. It is clear that proteins have to interact in a specific manner in order for messages/signals to be transmitted correctly. Therefore, significant efforts have been made to understand the driving mechanisms of protein-protein interactions [2]–[7]. The picture that has emerged from these studies illustrates the removal of non-polar residues from the aqueous environment as a major thermodynamic driving force for protein binding [8], [9]. Consistently, interaction surfaces have been shown to be enriched in hydrophobic residues, especially in the most buried regions of interfaces [10]–[12]. In contrast, specificity in interactions is believed to rely on shape complementarity, hydrogen bonding, and salt-bridge formation [13], [14]. In this context, the role of electrostatics in protein-protein interactions has been studied extensively [6], . It has been shown that salt bridges in protein interfaces can contribute favorably to protein stability and the free energy of binding through Coulombic interactions, but that this effect is often counterbalanced by very unfavorable desolvation [16]–[21]. Hence, the electrostatic component of the free energy of binding often destabilizes the protein complex. Despite that, salt bridges are still important for binding because of their contribution to interaction specificity [21]. This contribution is explained by the large energetic penalty for burying but not compensating for charged residues.

Some of the mechanisms and principals of protein-protein interactions derived from previous studies are likely to be challenged for interactions that involve intrinsically disordered (ID) segments of proteins [22]–[25]. One obvious reason is that ID segments lack a unique three-dimensional structure when free in solution and are likely to fluctuate between different conformations that lack any secondary structure or visit them only transiently [26], [27]. A few recent studies analyzed the interfaces of ID segments that are in complex with folded proteins [28]–[30]. In contrast to typical ID regions, which are generally enriched in charged residues and depleted in large hydrophobics, it was revealed that ID segments involved in protein binding tend to be enriched in hydrophobic residues [28]–[30]. Given the dominance of hydrophobic residues in ID segments that are part of interfaces, researchers have proposed that interactions mediated by ID segments may be less specific than interactions between folded proteins [29]. However, this idea seems to be at odds with results from various studies. For instance, intrinsic disorder has been shown to be important in specific protein-DNA interactions [31]. Specificity in interactions mediated by ID regions is often explained by the mechanism of coupled folding and binding, which gives ID binding regions the ability to mold into a precise fit for a given binding surface [32], [33]. However, there is a hint of paradoxical nature in this argument because the ability to mold implies the flexibility to fit a wide selection of binding surfaces promiscuously. Logic dictates that the sequence of the ID segment should encode determinants that constrain their promiscuity in binding. A reasonable source of specificity is the polar properties of ID segments.

Here, we devised a new structure-based computational method to identify ID segments that are in complex with other macromolecules. Making use of the extended nature of most ID segments when in complex with globular proteins, we identified complexes between globular proteins and ID segments (ID complexes henceforward) from the Protein Data Bank (PDB) by using radius-of-gyration-based selection criteria. The method was first benchmarked on 52 complexes where one partner is experimentally proven to be ID and then applied to a large non-redundant PDB dataset to identify new ID complexes. Consistent with previous studies, we find the interfaces of ID complexes to be enriched in hydrophobic residues, and that these residues contribute significantly to the stability of the interaction interfaces. However, our results show that polar interactions play a larger role in ID complexes than in structured protein complexes. Computational alanine scanning and salt-bridge analysis indicate that interfaces in ID complexes are highly complementary with respect to electrostatics, more so than interfaces of globular proteins. Follow-up calculations of the electrostatic contributions to the free energy of binding with DelPhi uncovered high desolvation penalties in ID complexes. However, these penalties are often nearly compensated by favourable Coulombic interactions that are significantly stronger than those in structured-structured protein complexes. In the light of the magnitude of the electrostatic energy terms that we estimated for ID complexes, we suggest that strong electrostatic interactions are a key component of the highly complementary interactions between ID segments and their partners that translates to high specificity.

## Results

### Identification of ID segments in complex with partner proteins

**Figure 1:**
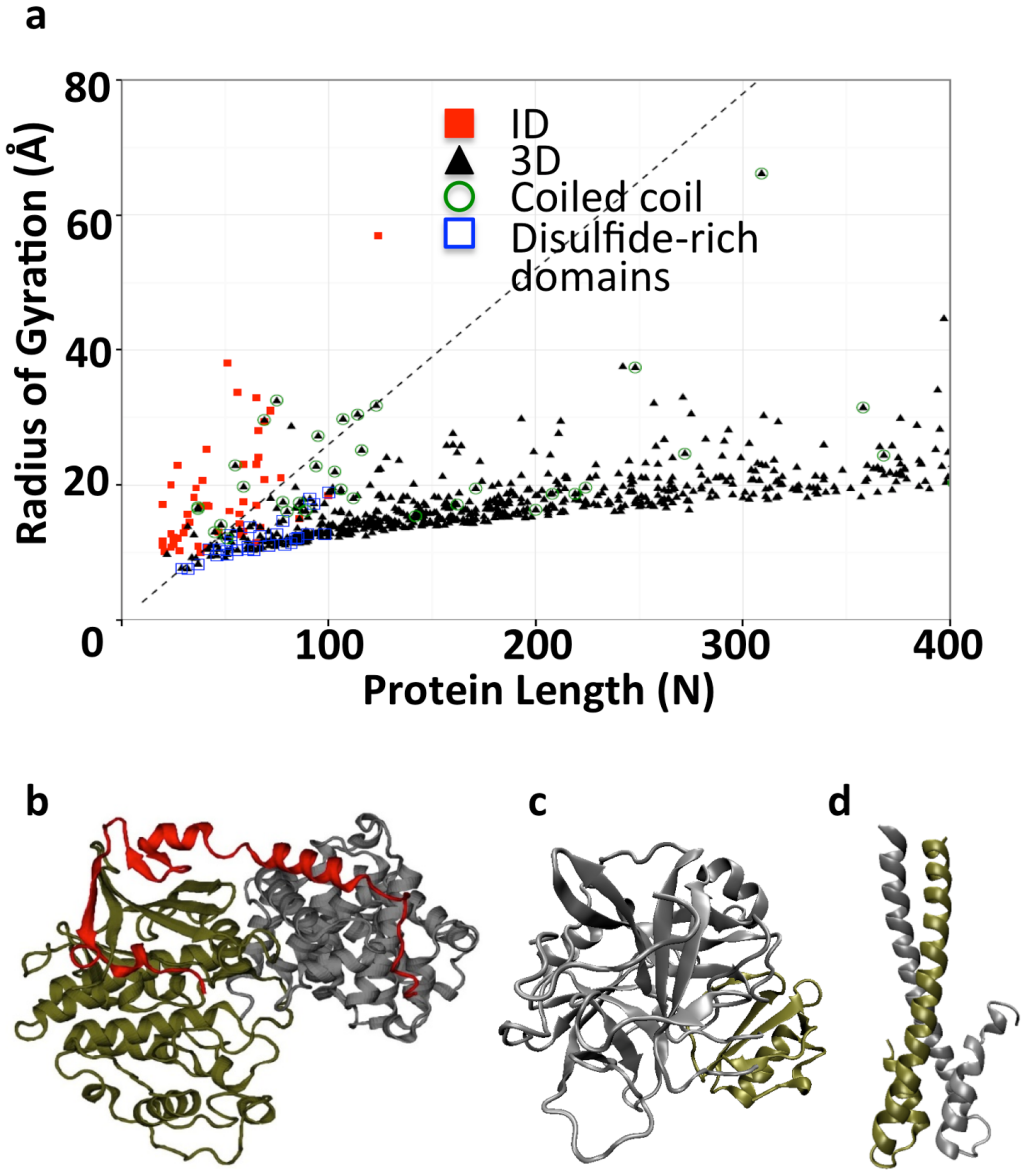
Radius of gyration (Rg) of interacting proteins. (a) Rg as a function of protein length for ID segments that interact with partner molecules (red squares, n = 52) and globular protein of the 3D complex dataset (black triangles, n = 762). 3D complex proteins that are disulfide-rich domains (n = 29) or coiled coils (n = 42) are enclosed in dark blue squares and green circles, respectively. The Rg/N threshold of 0.26 Å is represented by the dotted line. (b) Ribbon structure of the ID segment of p27 (red) that “wraps” around its complex partners cyclin A (grey) and Cdk2 (gold) (p27; PDB: 1JSU chain C, Rg = 21 Å, N = 69). (c) Ribbon structure of one of the 3D complexes, α-chymotrypsin (grey) and eglin c (gold) (bovine α-chymotrypsin; PDB: 1ACB chain E, Rg = 16 Å, N = 241). (d) Ribbon structure of the coiled coil EB1 (EB1; PDB: 1WU9 chain A, Rg = 20 Å, N = 59).

It has been shown that ID segments bury more solvent accessible surface area relative to their length than do structured proteins when they interact with other macromolecules [28]. In addition, several examples have been reported in which ID segments wrap around binding partners (e.g. p27Kip1 and p21Cip1 [34], [35], Figure 1b). This motivated us to hypothesize that ID segments could be identified based on their geometry when they are bound to structured proteins. Specifically, the radius of gyration (Rg) of a protein is a measure of its size and will reveal the extendedness of the protein chain when divided by chain length (N). We tested this hypothesis on a set of 52 long ID segments found in the literature for which the structure when in complex with a partner protein (mainly a globular one) is known. As a negative set, we selected 762 complexes from the 3D Complex database (Figure 1c), which is a database of proteins classified based on sequence, structure and topology. This should give a negative set that is enriched in structured proteins with different folds. We will refer to this dataset as the 3D complexes.

Consistent with our hypothesis and previous observations [30], we found that ID segments in complex with structured proteins tend to have larger Rg values for a given protein length than do structured proteins (Figure 1a). In order to see whether Rg/N can be used as an effective classifier of these structures, we used receiver operating characteristic (ROC) curves. The ROC curve constructed from calculating the Rg/N of the positive ID segment and the negative structured protein datasets has an area under the curve (AUC) of 0.986 (Figure S1a, Table S1).

However, Rg/N may not provide the best classifying results because Rg does not scale linearly with N. Flory has shown that for heteropolymers, Rg and N can be related by a simple scaling law [36]:
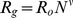
where R_o_ is a constant correlated with persistent length of the polymer and ν is a scaling factor that varies with different solvents due to resulting changes in the compaction of the proteins. Denatured proteins, which are in a collapsed state in poor solvent, have a scaling factor ν of 0.33 [36]. Figure S2 shows that the 3D complex structured proteins have, as expected [36], a low scaling factor ν of 0.35. On the other hand, the ν of the ID segments is 0.5 due to their expanded conformations. In order to determine whether a scaling factor ν different from 1 would improve the discrimination of ID segments from structured proteins, we constructed ROC curves at different scaling factors. We determined an optimal cutoff value at each scaling factor by selecting the threshold with the highest Matthew's correlation coefficient (MCC) [37], [38]. Out of all the scaling factors that we tested, ν of 1 results in a classifier that has the highest AUC (Table S1). At the chosen cutoff values, ν of 1 also gives the highest MCC with a low false discovery rate and high sensitivity. We cannot deny the possibility that there are other values of ν with greater performance. Nevertheless, our calculations clearly demonstrate that Rg/N is a very effective classifier for distinguishing extended ID segments from structured proteins when in complex with partner molecules.

Despite the excellent performance, we were interested in determining which proteins were falsely identified as ID. A close inspection of Figure 1a revealed that most of the false positives are short protein chains. Rg/N as a classifier appears to have difficulty discerning short ID segments from small, folded structures. Folded proteins shorter than 100 residues are often stabilized by disulfide bonds [39], which allow them to adopt relatively expanded conformations. Indeed, proteins with disulfide bonds are enriched among the false positives that we identified with the Rg/N classifier. Therefore, we removed proteins that are rich in disulfide bonds from the negative set. In addition, coiled coils often form long stretches of helical structure (Figure 1d). Although some coiled coils are known to be intrinsically disordered [40], this is not the case for all coiled coils. Consequently, coiled coils were also removed from the negative set. As a result of the exclusion of disulfide-rich complexes and coiled coils, the AUC rises slightly to 0.99 (Figure S1b, Table S2) and the MCC at an Rg/N of 0.26 Å (Figure S1c) is maximized to a value of 0.87±0.04. The false discovery rate and sensitivity at this threshold are 0.11 and 0.87 respectively.

Next, we applied the Rg/N classifier with a 0.26 Å cutoff on a non-redundant set of 6379 PDB files (see methods), which provided 330 potential ID complexes. Table 1 presents the list of datasets that we created and studied in this article.

**Table 1:** List of datasets analyzed and the number of structures in each dataset.

Dataset Name	Number of structures^a^
**Non-redundant PDB**	6918
**ID Complexes**	330
**High-resolution ID Complexes**	87 (68)
**3D Complexes**	762
**High-resolution 3D Complexes**	140 (109)

a The number of structures used in the continuum electrostatic calculations are in brackets.

### Validation of identified complexes

**Figure 2:**
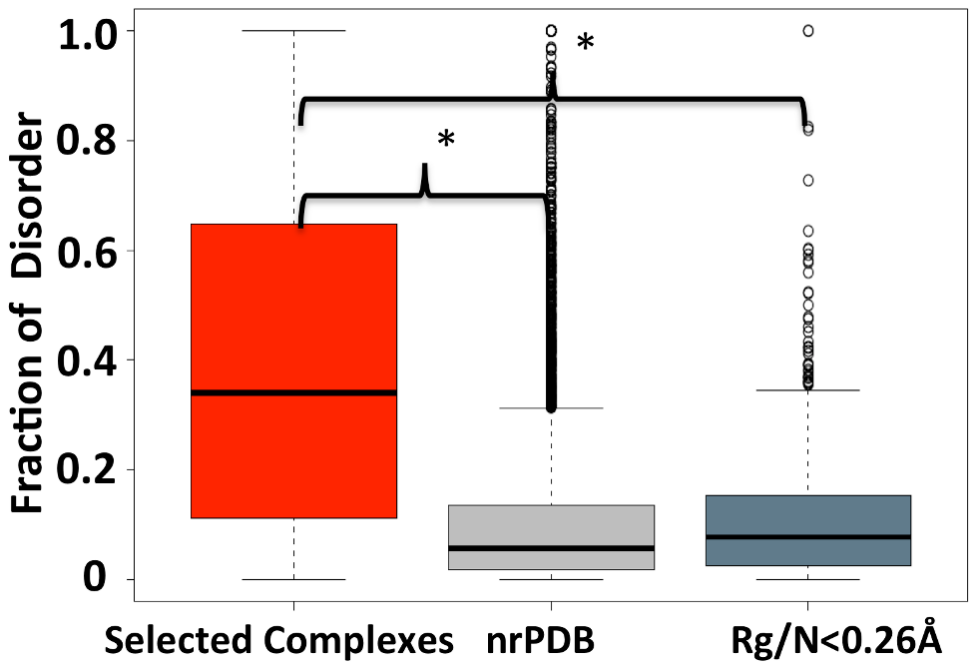
Box plot of the fraction of disordered residues in the selected ID complex dataset (Rg/N>0.26 Å) and two controls. Disorder content was calculated using Disopred2. The first control consists of all the structures in the non-redundant PDB dataset. The second control is the polypeptides of the non-redundant PDB dataset that have an Rg/N<0.26 Å while bound to a large protein partner. Asterisks identify distributions that are significantly different (*p* values<0.05; Wilcoxon test). Box plot identifies the middle 50% of the data, the median, and the extreme points. The entire set of data points is divided into quartiles and the inter-quartile range (IQR) is calculated as the difference between ×0.75 and ×0.25. The range of the 25% of the data points above (×0.75) and below (×0.25) the median (×0.50) is displayed as a filled box. The horizontal line represents the median. Data points greater or less than 1.5·IQR represent outliers and are shown as hollow circles.

A motivation for using a measure of geometry to select interacting ID segments of proteins is to have a method that does not rely on sequence information, which avoids biases in the analyses presented below. Sequence-based disorder predictions can instead be used to validate the selected structures. First, we predicted the disorder for the selected polypeptide chains with known coordinates (Figure 2). The selected chains have significantly higher predicted disorder content than a control set of non-redundant structures from the PDB (x˜_ID_ = 34%, x˜_nrPDB_ = 6%, *p* value = 7.8×10^−56^; Wilcoxon test, x˜ is the median). Moreover, the selected polypeptide chains are also predicted to be significantly more disordered when compared to chains that are not expanded and have Rg/N<0.26 Å (x˜_Rg/N<0.26 Å_ = 8%, *p* value = 8.1×10^−42^; Wilcoxon test).

Nevertheless, there are still sequences in the identified dataset that have low intrinsic disorder predicted based on their primary structure. The lower than expected predicted disorder may be explainable by the enrichment of ID interaction regions in the selected sequences. Interaction-prone regions within ID segments are known to be highly hydrophobic and have residue compositions more similar to the buried regions of structured proteins than to the rest of the ID segments [29]. To test this reasoning, we extended the sequence of the identified ID segments by 30 residues at their N and C-termini (i.e. by residues lacking coordinates in PDB files) and repeated the analysis. As expected, the distribution of percentage disorder increases significantly (Figure S3, x˜_ID_ = 34%, x˜_extended ID_ = 41%, *p* value = 8.1×10^−3^; Wilcoxon test), which suggests that the flanking regions of these ID segments are often more disordered than the interacting regions. As a considerable number of ID segments in our set do not have both flanking regions, i.e., they are at the termini, or the extended sequences were not readily found, we expect the difference to be greater still if we included more flanking regions.

**Figure 3:**
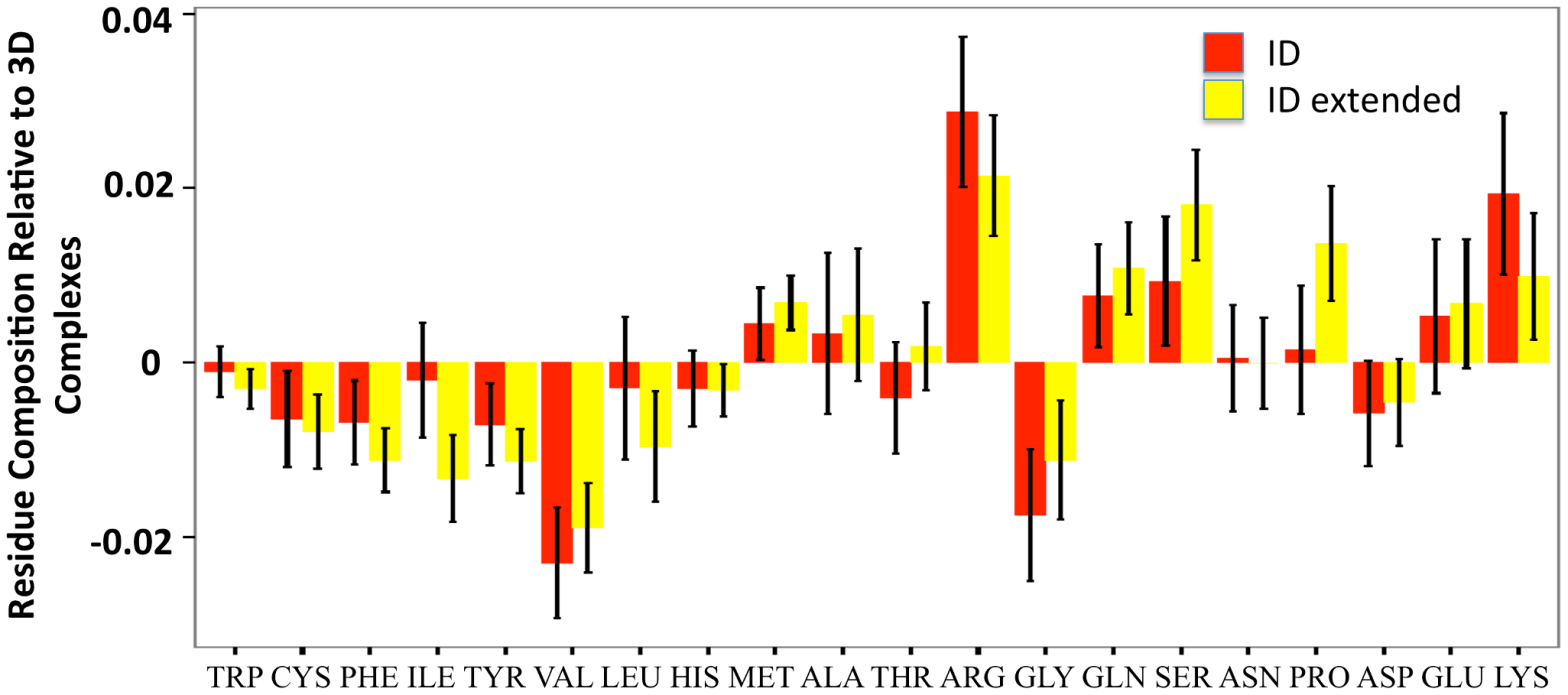
Residue composition of the proteins in the selected ID set relative to the 3D complex dataset. Averaged percentage residue compositions from ID dataset are subtracted by the respective percentage from the 3D complex. Positive and negative values indicate an enrichment and depletion, respectively, of a specific residue in the ID complex set with respect to the 3D complex dataset. Amino acids are sorted according to their ranking in protein chain flexibility [41], [80]. The residue composition for the ID segments (only residues with coordinates in the PDB) and extended ID segments (30 residues on each end) are shown in red and yellow, respectively.

The overall amino acid composition of the ID segments, which includes only residues with coordinates in the PDB files, is in agreement with the disorder prediction. The composition of the identified protein segments shown in Figure 3 is enriched in disorder-promoting residues compared to 3D complex proteins [41], especially charged residues such as R and K (R *p* value = 4.2×10^−9^, K *p* value = 1.4×10^−4^; Wilcoxon Test). The order-promoting residues, which are generally the hydrophobic amino acids, are depleted in our dataset (W *p* value = 2.6×10^−13^, F *p* value = 3.7×10^−8^, I *p* value = 4.5×10^−3^, L *p* value = 7.7×10^−2^, V *p* value = 9.1×10^−29^, Y *p* value = 1.9×10^−11^; Wilcoxon Test). When we again extended the number of residues analyzed by 30 on each end of the selected polypeptide chains, there was an increase in some of the disorder promoting residues (Figure 3). In order to put the residue composition of the ID segments that we identified into perspective, Figure S4 also shows the relative residue composition for the set of 52 ID segments from our literature search that we used to evaluate the classifier as well as 1150 ID protein segments taken from the DisProt database, which is a database of proteins with experimental evidence for intrinsic disorder [42]. Although the exact magnitude of the enrichment or depletion of specific amino acids differs, order-promoting hydrophobic residues are depleted and disorder-promoting charged and polar residues are enriched in all ID protein datasets when compared to 3D complex proteins. In summary, the presented results show that our method is able to select for ID proteins, and respectively, for ID protein segments that are in complex with other macromolecules.

Another known property of proteins with ID segments is their involvement in signalling and regulation [43]. By classifying gene ontology terms into nine major groups and assigning the ID segments into these groups, we observed significant enrichment of proteins involved in cytoskeleton, signalling, and transport (Figure S5a). We also observed that our ID dataset is not biased towards a single group of proteins (Figure S5b).

### Characterization of complexes

The main focus of our study is on the interaction between ID segments and their structured binding targets, so we analyzed the interface residue composition. Interface residues are categorized into the rim and core as defined by Levy [10]. The residues in the interface core are the most buried residues upon protein binding and are generally at the central region of the interface. The residues on the outer edges of the interface that remain partially exposed to solvent are part of the interface rim. For complexes between globular proteins, it has been shown that the interface rim defined in this way has a residue composition similar to the protein surface [10]. In contrast, the core has a distinctive residue composition that is an intermediate between the surface and interior of folded proteins.

**Figure 4:**
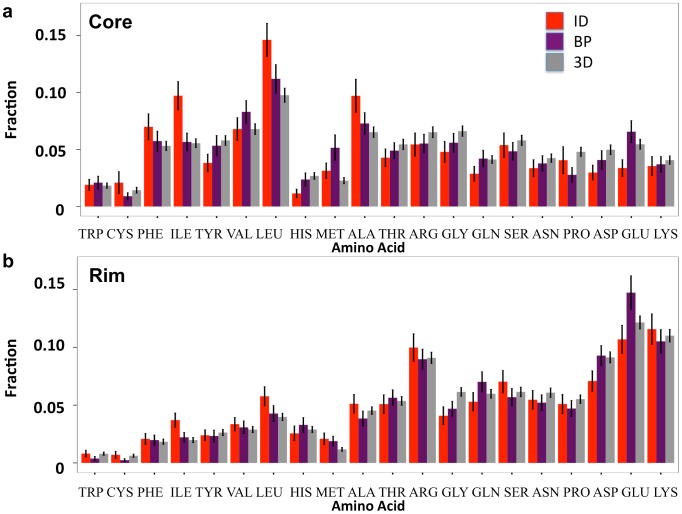
Interface residue composition. (a) The residue composition at the core regions of complex interfaces. (b) The residue composition at the rim regions of complex interfaces. The interface residue compositions of ID segments, ID binding partners (BPs), and 3D complex proteins are shown in red, magenta, and grey respectively.

Figure 4 shows that both ID and 3D complexes are significantly enriched in hydrophobic residues in the core interface regions when compared to the rim. The extent of enrichment in hydrophobic residues is especially notable in the core region of the ID segment (e.g. ID core vs. ID rim: W *p* value = 1.0×10^−4^, F *p* value = 3.4×10^−13^, I *p* value = 2.3×10^−14^, Y *p* value = 0.16, V *p* value = 1.4×10^−6^, L *p* value = 1.2×10^−19^, A *p* value = 1.8×10^−6^; Wilcoxon test). Importantly, the core residues of ID segments are more often hydrophobic than the core residues in 3D complexes. Compared to the 3D interface cores, the ID segment cores have lower percentage composition of charged and polar residues (ID core vs. 3D core: K *p* value = 7.5×10^−3^, E *p* value = 2.6×10^−7^, D *p* value = 7.4×10^−7^; R *p* value = 6.1×10^−3^, H *p* value = 7.9×10^−8^, N *p* value = 5.5×10^−3^, Q *p* value = 9.3×10^−5^; Wilcoxon test). Instead, there are greater proportions of hydrophobic residues such as F, I, L and A (ID core vs. 3D core: F *p* value = 5.8×10^−2^, I *p* value = 9.0×10^−11^, L *p* value = 5.7×10^−11^, A *p* value = 1.0×10^−4^; Wilcoxon test). These findings advocate that hydrophobic residues play a critical role in ID complex interfaces, which is in agreement with previous studies by Vacic et al. and Mészáros et al. [29], [30].

However, comparison of Figure 4a and 4b clearly reveals that the rim contains significantly more charged and polar residues than the core and this is the case not only for 3D complexes, as shown previously [10], but also for ID complexes. Compared to the core, K, E, D, N, R and H are significantly enriched in the rim of ID segments (ID core vs. ID rim: K *p* value = 4.7×10^−26^, E *p* value = 8.2×10^−24^, D *p* value = 3.4×10^−14^, N *p* value = 8.6×10^−7^, R *p* value = 3.8×10^−11^, H *p* value = 2.2×10^−4^; Wilcoxon test). These distinctions are evidence for the good efficacy of Levy's interface definitions. In contrast to the interface core, we find less significant differences in the distribution of residues in the rim when comparing ID and 3D complexes.

Accounting for the total size of the interface core and rim regions provides another perspective for understanding the differences between ID and 3D complexes (Table S3). One very distinctive feature is the large number of residues in the rim of binding partners of ID segments (BPs). On average, the rim of BPs includes 12% more residues than the rim in classical protein complexes (*p* value = 6.6×10^−8^; Wilcoxon test) while the number of residues on the rim of the ID segment is closer to that of 3D complex proteins (*p* value = 0.71; Wilcoxon test). As a result, the average number of most charged residue types in the rim is significantly greater in the rim of BPs compared to the rim of ID segments (E *p* value = 1.1×10^−8^, D *p* value = 1.4×10^−7^, H *p* value = 8.8×10^−3^; Wilcoxon test) as well as the rim of 3D complexes (E *p* value = 8.6×10^−6^, R *p* value = 1.7×10^−2^, D *p* value = 3.7×10^−3^; Wilcoxon test) (Figure S6). The interface core of ID segments, on the other hand, contains 17% and 22% fewer residues compared to the BPs' and 3D complex proteins' interface core, respectively (ID vs. ID partner *p* value = 4.8×10^−6^, ID vs. 3D *p* value = 2.2×10^−3^; Wilcoxon test). In summary, the ID complex interface appears to consist of a small but very hydrophobic core on the ID segment's side and a large and polar rim on the BP's side.

To gain further insights into the interactions in ID and 3D complexes, we calculated the number of salt bridges and hydrogen bonds (Table 2). An average of 48% more salt bridges are found in ID complexes compared to complexes with only structured members (*p* value = 5.0×10^−3^; Wilcoxon test). This difference is still present after normalization by the average interface area, though not statistically significant. There are also slightly more hydrogen bonds, by 13% on average, in ID complex interfaces (*p* value = 0.082; Wilcoxon test). The numbers of hydrogen bonds in ID complexes is comparable when we normalize by interface areas. Overall, these analyses confirm that the core of ID interfaces are highly enriched in hydrophobic residues, but they also reveal that charged residues are abundant in the rim, especially on the BP, and are involved in salt bridge or hydrogen bond formation.

**Table 2:** Interface characteristics.

	ID Complexes	3D Complexes	*p* values^c^
**Hydrogen bonds** a	12.01	10.64	0.082
**Per 100 Å^2^**	0.85	0.92	0.32
**Salt-bridges** a	6.68	4.51	5.0×10^−3^
**Per 100 Å^2^**	0.48	0.44	0.15
**Interface SASA** b	1411.1	1148.4	9.6×10^−5^

a Average number of hydrogen bonds or salt bridges per complex interface.

b Average SASA buried in the interface (1/2 of the sum of 2 sides).

c Significance of the difference between ID and 3D complexes (Wilcoxon test).

### Computational alanine scanning analysis

To analyze the contributions of individual residues to the stability of the interaction interface, we carried out computational alanine scans. It is well understood that, in general, only a small number of interface residues, called “hot spots”, are making the essential interactions. We defined hot spot residues as those which have a ΔΔG_bind_ of >1.5 kcal/mol when mutated into alanine. In order to avoid artefacts due to low-resolution data, we performed the ALA-scan on subsets of the ID and 3D complexes that contain only high-resolution crystal structures (resolution <2.5 Å; see methods). We also analyzed our results through comparisons with the 3D complex proteins to minimize any bias in the calculations. Moreover, we analyzed the effect of alanine mutations for residues in the ID segments and their BP separately.

**Figure 5:**
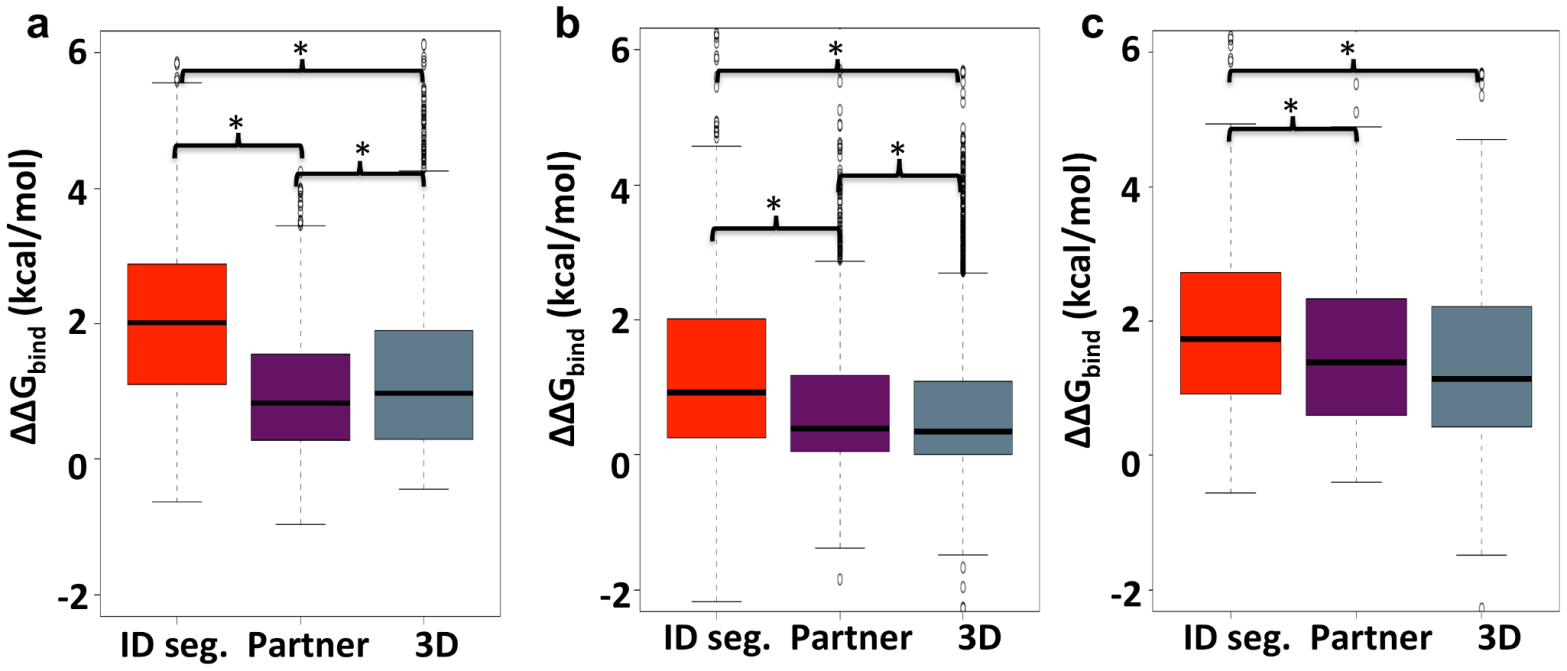
Box plots of changes in free energy of binding (ΔΔG_bind_) in the alanine scan. Free energy changes for hydrophobic residues, charged residues, and only charged residues that are forming salt bridge interactions are shown in (a), (b) and (c), respectively. Asterisks identify distributions that are significantly different (*p* values<0.05; Wilcoxon test). The results for residues in the ID segments, ID binding partners (BPs), and 3D complex proteins are shown in red, magenta and grey respectively.

Analysis of our high-resolution datasets reveals a greater percentage of interface residues qualifying as hot spots in ID complexes than in 3D complex proteins at 27% and 20%, respectively (*p* value = 1.3×10^−7^; Wilcoxon test). The percentage of interface residues qualifying as hot spots averages at 40% and 21% (*p* value = 4.4×10^−21^; Wilcoxon test) for the ID segment and their BPs respectively. Next, we dissected the contribution of hydrophobic and charged residues to the interaction energy. Figure 5a shows the distribution of change in binding free energies (ΔΔG_bind_) for the mutation of hydrophobic residues (V, L, I, F, M, Y, W) to alanine in ID complexes and 3D complexes. ALA mutations of hydrophobic residues in ID segments are generally more destabilizing than ALA mutations of hydrophobic residues in 3D complexes (x˜_ID_ = 2.0 kcal/mol, x˜_3D_ = 0.97 kcal/mol, *p* value = 1.8×10^−59^; Wilcoxon test). This difference may be expected given the enrichment for large hydrophobic residues with large surface area in the interface core of ID segments. Another possible explanation may be that the packing of the side chains in the interface is better compared to the rigid binding in structured proteins. A previous study has shown a greater number of atoms in contact per residue in ID complex interfaces [30], which could give rise to greater interaction energy for non-polar residues. Regardless of the reason, the fact that our results show higher ΔΔG_bind_ for the hydrophobic residues in ID segments once again confirms that hydrophobic interactions are a key driving force for ID segment binding.

A more complex picture emerges when analyzing the effect of mutations of charged residues. Figures 5b and 5c reveal the change in binding free energies upon alanine mutations of charged residues (E, D, R, K, H) and only those charged residues that are involved in salt bridges, respectively. As expected, the comparison of the ALA-scan results for these two groups shows that the ion-pairing residues tend to have much higher ΔΔG_bind_ (x˜_charged_ = 0.43 kcal/mol, x˜_salt bridge_ = 1.32 kcal/mol). The results of the ALA-scan for all charged residues (Figure 5b) demonstrate that mutations of charged residues on the ID segment side of the interface are, on average, significantly more destabilizing than ALA mutations of charged residues in 3D complexes (x˜_ID_ = 0.93 kcal/mol, x˜_3D_ = 0.35 kcal/mol, *p* value = 1.9×10^−28^; Wilcoxon test). Moreover, mutations of these charged interface residues in the ID segment result in significantly higher ΔΔG_bind_ when compared to mutations of charged residues on the BP's side (x˜_ID_ = 0.93 kcal/mol, x˜_BP_ = 0.39 kcal/mol, *p* value = 4.3×10^−18^; Wilcoxon Test). This finding suggests that a greater proportion of the charged residues on the ID segment's interface are making specific interactions than the charged residues on the partner protein. Indeed, the disparity in ΔΔG_bind_ for charged residues in the ID segments or their BP is smaller, though still significantly different (x˜_ID_ = 1.7 kcal/mol, x˜_BP_ = 1.4 kcal/mol, *p* value 6.6×10^−3^; Wilcoxon Test), when we analyzed only the salt-bridge-forming residues (Figure 5c). Importantly, the ALA mutations of salt-bridging residues in ID segments are significantly more destabilizing than ALA mutations of salt-bridging residues in 3D complexes (x˜_ID_ = 1.7 kcal/mol, x˜_3D_ = 1.1 kcal/mol, *p* value = 9.0×10^−7^; Wilcoxon Test).

As pointed out above, there is a relationship between change in solvent accessible surface area and ΔΔG_bind_, and this is explored in Figure S7. The correlation between ΔΔG_bind_ and change in accessible surface area is, as expected, strong for hydrophobic residues (R^2^_ID_ = 0.67, R^2^_BP_ = 0.62) but extremely weak for charged residues (R^2^_ID_ = 0.25, R^2^_BP_ = 0.16) and those that form salt bridges (R^2^_ID_ = 0.13, Figure S7a). The correlation is worst for the bridging residues on the BPs' side of interfaces (R^2^_BP_ = 0.036) because these residues often have small changes in solvent accessible surface area upon binding as part of the partially solvent-exposed regions. Hence, normalizing ΔΔG_bind_ by the change in solvent accessible surface area has a pronounced effect for hydrophobic residues (Figure S7b versus Figure 5a), but less so for charged residues (Figure S7c versus Figure 5b). Indeed, the normalized ΔΔG_bind_ of charged residues in ID segments are still significantly larger than those in 3D complexes (x˜_ID_ = 0.019 kcal/mol**Å^2^**, x˜_3D_ = 0.013 kcal/mol**Å^2^**, *p* value = 7.8×10^−9^; Wilcoxon Test) (Figure S7c).

It is clear that the results of single mutation experiments involving charged residues do not reveal whether their polar interactions are stabilizing or destabilizing. The results would only reflect the effect of the removal of this particular charged residue on the binding affinity [20], [21]. Moreover, charged residues on the proteins can appear to have weak complementarity across the complex interface while significant complementarity can still be found in their electrostatic potential [15]. Hence, ALA-scan results cannot be used to compare the importance of electrostatic interactions for the stability of ID and 3D complexes.

### Role of electrostatics in ID segment-partner interactions

Consequently, we used DelPhi for continuum electrostatic calculations in order to get more accurate insights into the contribution of electrostatics to the binding of ID segments to partner proteins. To estimate the electrostatic free energies of binding, we used the same approach that has previously been used to study the binding of folded proteins [20]. This approach assumes that the structure of both binding partners do not change upon complexation. It has to be stressed that this is a considerably big assumption to make, particularly in the case of ID segments that often fold upon binding (see also below). However, Sheinerman and Honig previously outlined how calculations based on this assumption can be used to compare the electrostatic contributions to binding between different protein complexes [20]. Consistently, we did not directly compare the results with experimental data but compared the electrostatic contributions to the interface stability of ID and 3D complexes.

**Figure 6:**
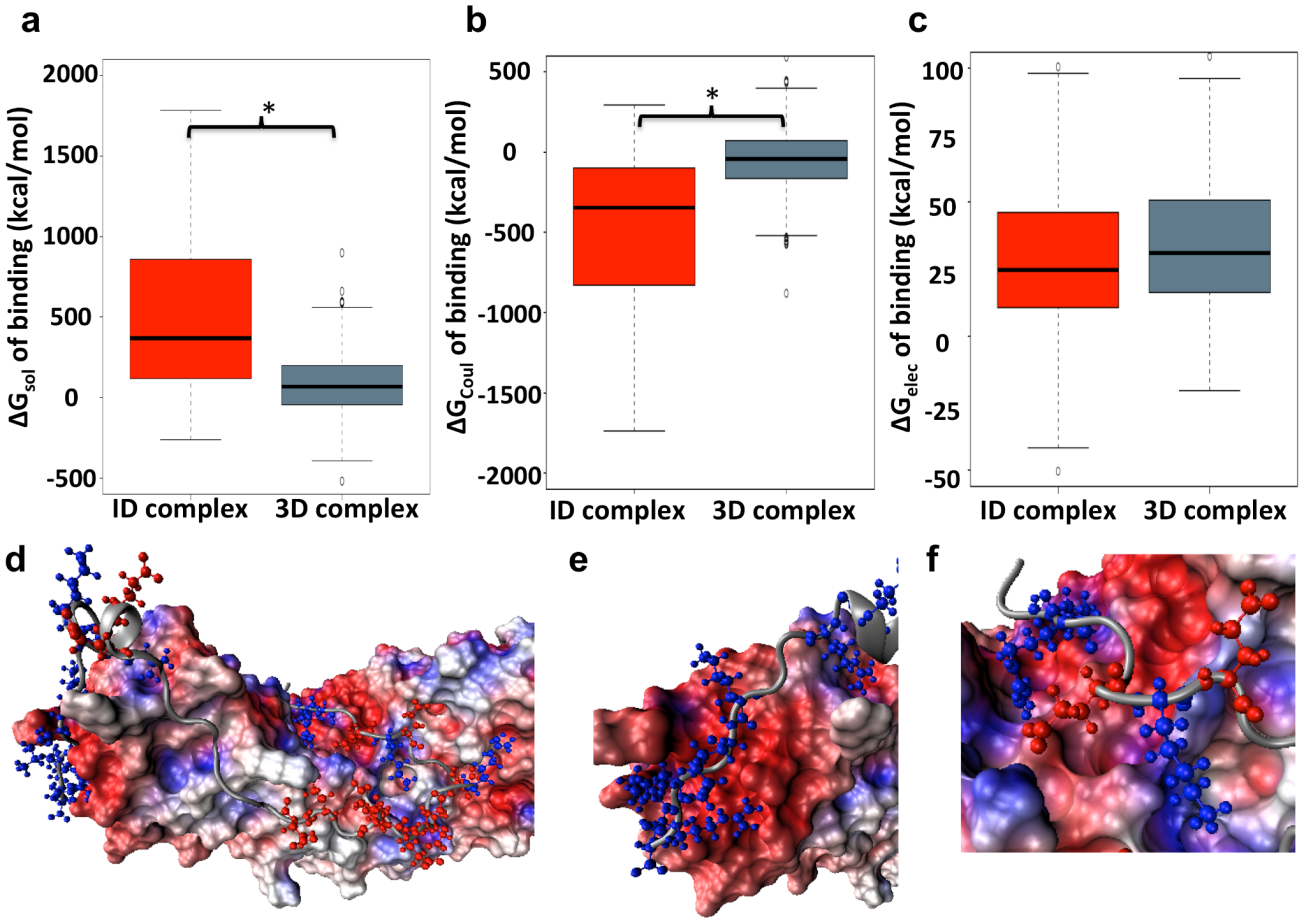
Box plots of electrostatic components of the binding free energy. (a) Electrostatic contribution to the desolvation free energy of binding. (b) Coulombic interaction energy of binding. (c) Total electrostatic free energy of binding. Asterisks identify distributions that are significantly different (*p* values<0.05; Wilcoxon test). Electrostatic contributions to binding are shown for ID complexes and 3D complexes in red and grey, respectively. (d–f) Nup50/importin-α2 is an example of a complex that involves burial of extensive polar surfaces (PDB: 2C1M [81]). The surface of importin-α2 was generated using a probe radius of 1.5 Å. The surface is colored using the electrostatic potential map of importin-α2 that was generated by Delphi. The ID segment, Nup50, is represented by the cartoon ribbon structure with ARG, LYS, and HIS residues colored in blue and GLU and ASP residues colored in red. (d) The full view of the interacting region of the Nup50/importin-α2 complex. (e) Nup50 (37–46) contains a high concentration of positively charged residues that bind to an acidic region of importin-α2. (f) The positively charged residues of the N-terminus of Nup50 are complementary to an acidic surface on its binding partner.

The distribution of the polar-desolvation energies of binding is shown in Figure 6a. As expected, the electrostatic desolvation energy of binding is higher for ID complexes than for 3D complexes (x˜_ID_ = 368.11 kcal/mol, x˜_3D_ = 68.59 kcal/mol, *p* value = 2.2×10^−10^; Wilcoxon test). The high electrostatic desolvation energies reflect the cost of removing the polar surfaces of the larger ID complex interfaces from the high dielectric environment of the polar solvent and burying them within the low dielectric protein environment. It is interesting to note that, while most complexes have very unfavorable desolvation energies of binding, they are favorable for 32% of 3D complexes. Favorable polar solvation free energies of binding are less intuitive but were observed previously [44].

Unfavorable polar desolvation may be compensated by inter-chain electrostatic interactions, such as salt bridges. Indeed, the contribution of the Coulombic energies of binding in ID complexes is generally high enough to overcome most of the high polar-desolvation energies. Figure 6b shows that Coulombic contributions to binding are also greater in ID complexes than in conventional complexes (x˜_ID_ = −346.92 kcal/mol, x˜_3D_ = −42.86 kcal/mol, *p* value 1.3×10^−10^; Wilcoxon test). Taken together, the sum of solvation free energy and the Coulombic energy result in a total contribution of electrostatics to binding that is, on average, not favorable and very similar in ID complexes and 3D complexes (x˜_ID_ = 24.64 kcal/mol, x˜_3D_ = 31.02 kcal/mol, *p* value = 0.13; Wilcoxon test; Figure 6c). These findings are also confirmed in a subset of the ID complexes with literature support that we used to train the classifier (Figure S8). Furthermore, these trends hold true even upon normalization of the binding energy components by the interface areas (Figure S9).

## Discussion

We now know that proteins with ID segments are prevalent and have important biological roles [45], [46]. Short motifs, or molecular recognition features (MoRFs), within ID segments often mediate interactions involving proteins. A few studies have recently analyzed the interfaces of ID segments that are in complex with folded proteins [28]–[30] in order to reveal the driving forces between the interactions of ID segments and their partners. Statistical analyses revealed that they are more hydrophobic than interfaces of globular proteins and have more hydrophobic-hydrophobic interactions than polar-polar interactions. We would like to stress that our analysis confirms that the interface cores formed by ID segments are enriched in hydrophobic residues, and that *in silico* mutations of these hydrophobic residues to ALA often result in a larger ΔΔG_bind_ when compared to classical protein complexes. Although the latter finding has to be interpreted with care (see below), it suggests that hydrophobic residues in ID segments contribute significantly to the binding affinity of ID complexes. However, the rim regions are rich in charged and polar residues just like the rim regions of complexes formed between globular proteins. Moreover, the fact that the rims of BPs contain a 19% greater total number of residues than the rim of the ID segments contributes to a 33% greater average number of charged residues in the rim of BPs. An adequate complementation of these charges in the rim of the BPs by fewer charges in the smaller rim of the ID segments would require a strategic placement of the latter.

Hence we asked the question: how important are the charged residues on either side of the ID complex interface? It is clear that statistical analyses of residue composition enable only indirect conclusions on the energetics that govern protein interactions. Therefore, we conducted a systematic ALA-scan of charged residues and determined electrostatic contributions to the free energy of binding with DelPhi. The ALA-scan revealed that, generally, mutating a charged interface residue in an ID segment destabilized the complex more than the corresponding mutations in the BP or mutating a charged residue in a complex of globular proteins. Put into the context of larger rims found for BPs, these results could mean that removing a single charged residue in the rim of the ID segment often breaks interactions with multiple charged residues on the BP's side. In the interpretation of these results, three caveats have to be taken into account: (i) single mutation experiments involving charged residues do not reveal whether the polar interactions are stabilizing or destabilizing the complex, (ii) FoldX was trained mainly on structured proteins [47], and (iii) calculations do not take into account the conformational changes upon binding. The latter two most likely lead to an overestimation of ΔΔG_bind_ for ID complexes. Mutations in ID segments often demonstrate strong enthalpy-entropy compensations [48] that are unlikely to be fully accounted for by FoldX. Nevertheless, computational alanine scanning does provide insights into the energetics of the immediate interactions side chains make at an interface [49]. Therefore our results strongly suggest that charged residues in ID segments make more complementary interactions with their partners than do charged residues in 3D complexes, which is consistent with our salt-bridge analysis, and that these interactions are potentially optimized. We found several examples in the literature confirming that mutations of charged residues in ID segments can have significant effects on binding affinities. For instance, the complex of the kinase-inducible domain of CREB with the KIX domain of CBP can be destabilized by reversing the charges of key residues, and the stability can be partially rescued by reversing the charge of the complementary residue across the interface [50]. Another example involves WASP, whose dissociation constant with Cdc42 has been shown to increase from 74 nM to 2.6 µM following mutation of 3 consecutive lysines to alanines [51]. Lastly, a comparison of the sequence of the D2 domains of p21 and p27 revealed four glutamate residues in p27 that are substituted by one alanine, two arginines and one lysine in p21. These differences have been proposed to be responsible for a drop in affinity from 70 nM to 5.3 µM [35]. These examples support the notion that charged residues in ID segments can form highly complementary interactions at interfaces. Together with our findings, it provides evidence that points to strong, charged interactions as a possible origin of the high specificity of ID segment interaction.

In order to corroborate this hypothesis, we investigated electrostatic complementarity using Delphi. These calculations revealed that binding of ID segments involves strong Coulombic interactions. The magnitude of the Coulombic binding energy is significantly higher than in structured protein complexes, but the desolvation penalty generally dominates and produces a net destabilizing effect. Nevertheless, electrostatics is still likely to play an important role in the specificity of ID segment binding. What is important for the specificity is the balance between the polar-desolvation energy and the Coulombic energy. Non-complementary charged groups will cause protein interactions to become unfavorable by not paying the penalties for being buried in a low dielectric environment. Moreover, it is highly likely that the electrostatic complementarity that we found affects association kinetics [52], [53], which would also have a significant impact on the recognition of cognate and non-cognate partners [54]. Importantly, the difference in Coulombic free energy of binding between ID and conventional protein complexes shows that ID complexes are clearly in a different category in regards to protein-protein interactions (Figure 6 d–f illustrate the extent of electrostatic complementarity in one ID complex).

It is clear that the approach we used here to assess electrostatics, which assumes that both binding partners do not change their structure upon complexation, is highly oversimplifying the problem, particularly for ID segments. The free energy changes associated with the structural changes necessary for or induced by binding (ΔG_strain_) are always positive. Hence our estimates of the electrostatic free energy change upon binding are too low. In the case where the ID segments folds upon binding, ΔG_strain_ may be significant. However, it has been proposed that many ID segments are bound via conformation selection, i.e. a pre-existing conformation is selected during binding out of the population of conformations sampled by the ID segment [26], [55]. In cases where the binding-competent conformation is sampled frequently, ΔG_strain_ may be comparable to the one of interacting globular monomers. In this context it is interesting to note that Massova and Kollman calculated the binding free energies for the interaction between Mdm2 and an ID segment of p53 that adopts a helical conformation in the complex [56]. Their approach included calculations of the electrostatic contribution to binding that are very similar to the ones used here. Most importantly, despite not treating the folding of p53 into a helix explicitly in their calculations, good agreements with experimental affinities were found.

Hence, the uncertainty in our estimates deserves attention but may not be systematically greater for the ID complexes than the 3D complexes. The large magnitude of the differences between the electrostatic free energy of binding of the ID complexes and structured protein complexes, which is also consistent with results from our salt-bridge and ALA-scan analysis, give credence to our hypothesis of the distinct importance of electrostatic interactions in ID complexes.

### Conclusion

In contrast to protein folding, interactions between proteins are more strongly driven by polar interactions. As pointed out by Sheinerman and colleagues [6], protein folding is largely driven by the burial of large hydrophobic areas, which is necessary to offset the entropic cost of folding. In the binding of folded monomers, entropic penalties are much smaller, which reduces the requirements for compensatory energy gains upon binding, for instance, through the burial of large hydrophobic areas. The binding mechanism of ID segments can be considered as an intermediate between protein folding and the interaction between folded proteins in terms of entropic costs. This line of reasoning explains why ID complex stability must be afforded by the burial of extensive hydrophobic surfaces. Therefore, the interface size and packing of hydrophobic residues are key factors in determining complex affinity. The enrichment of hydrophobic residues others [28], [29] and we found in the interface of ID complexes is a testimony of this mechanism. However, protein binding of large ID segments in extended conformations involves the formation of extensive interface-rim regions that are only partially accessible to solvent. These large, polar interface areas can cause high desolvation costs that have to be offset by complementary electrostatic interactions. In this way, the ID segments can only bind to specific binding partner(s) with sufficient electrostatic complementarity. This may be the key for interacting with multiple partners while being specific to every one of them. Hence, packing and size of the interface as well as its electrostatic complementarity are partners in the fine-tuning of the affinity and selectivity of ID segment interactions [22], [33], [57].

## Methods

### 3D complex and ID complex test dataset

The structured protein dataset was derived from the 3D Complex database [58]. 3D Complex is a database of protein complexes classified by their known three-dimensional structure in a hierarchical way. We selected dimers out of the Quaternary Structure Families of complexes and downloaded the structure coordinates from the Protein Data Bank (PDB) [59]. The Quaternary Structure Families grouping has little to no relation to the sequence identity of the proteins. Therefore, we applied a sequence alignment and clustering procedure on the dataset to remove redundancies. We used EMBOSS Needle [60], a pairwise alignment tool, with the scoring matrix EBLOSUM62 and default gap penalties. Needle outputs sequence percentage similarity and identity for each chain pair. We defined redundant sequences using a sequence identity threshold defined by Rost [61]:
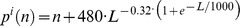
where n is the number of percentage points above the default curve and *L* is the protein length. Using a threshold determined at n = 3 returned 782 non-redundant structures. With protein chains that are shorter than 20 residues removed, a final dataset of 762 protein chains was created.

The structures for the test set of ID complexes, i.e. protein complexes with one interaction partner that has been identified as intrinsically disordered in experiments, were selected through literature search. We identified 40 ID complexes by searching primary literature. We further extended the dataset by including complexes identified by Mészáros and coworkers, which resulted in a total of 74 complexes [30], [62]. Sequence alignment and clustering reduced the final test set of ID complexes to 52.

### Classifier selection

The radius of gyration (Rg) was calculated using the Perl script rgyr.pl from the MMTSB tool set [63]. It is defined as:
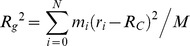
where the position of the atom *i* and the center of mass are 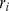 and 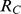 respectively. The mass of atom *i* is 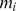 and the total mass is *M*. Rg/N was calculated for the α-carbon coordinates of the proteins.

The performance of Rg/N for separating the structures of the ID complex test set from those of the 3D complex set was first evaluated using Receiver Operating Characteristic (ROC) curves. Hence, the ID complex test set and 3D complex dataset were used as the positive and negative datasets, respectively. The area under the ROC curve (AUC) was used for evaluating the ROC curves. AUC is correlated to the accuracy of the classifier and has the advantage of being insensitive to class skew [64].

However, the ROC curve does not readily identify the optimal Rg/N threshold. A Matthew's correlation coefficient (MCC) curve was then used to identify the optimal threshold of Rg/N [38]. The MCC is defined as:
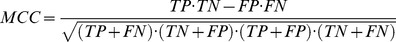
where *TP* is the true positive, *TN* is the true negative, *FP* is the false positive, and *FN* is the false negative. The Rg/N of 0.26 Å was selected as the optimal threshold for identifying ID complexes. To get an averaged ROC curve and confidence levels for the MCC curve, we randomly sampled half of the ID and 3D complex datasets for 1000 repetitions. The threshold averaged ROC curves and the averaged MCC curve were calculated by using the ROCR package [65].

### Identification of new ID complexes

In order to identify new ID complexes with the Rg/N classifier, a list of non-redundant PDBs was taken from NCBI nr table (ftp:/ftp.ncbi.nlm.nih.gov/mmdb/nrtable/) [66]. The nrlevel 0 was chosen, which means sequences were grouped using BLAST *p* values of 10^−7^ as a cutoff. We applied the classifier only on protein chains that have more than 20 residues with coordinates present and are interacting with at least one other partner in the PDB file. At least one of the interacting chains also has to have more than 70 residues. Protein structures from the 3D complex set that overlap in their Rg/N with structures from the ID complex test set (Figure 1a) are often coiled coils or disulfide-rich domains. Therefore coiled coils and disulfide-rich domains were removed from the non-redundant protein dataset. We used Socket [67] to identify coiled coils. Socket is a program that identifies coiled coils by recognizing the ‘knobs-into-holes’ packing patterns formed by interlocking α-helices in PDB file structures. Disulfide-rich protein domains are small domains whose structures are stabilized by disulfide bonds. These stable protein domains tend to be shorter than 100 residues [39]. Consequently, all protein chains shorter than 100 residues with two or more disulfide bonds were removed. Finally, transmembrane proteins were also removed because they also have high Rg/N. We used the HMMTOP server for the prediction of transmembrane helices [68].

### Sequence-based disorder prediction

Disopred2 [45] was used to predict disorder from protein sequence. Intrinsic disorder content for protein segments of interest (e.g., segments with coordinates) was extracted from predictions for the entire protein. Whenever possible, the complete native protein sequence from the Uniprot database was used for the prediction. Otherwise, the full protein chain sequence from the PDB file was used.

### Gene ontology analysis

Proteins were mapped to gene ontology terms using the Gene Ontology (GO) database [69]. The gene association file for Protein Data Bank Ids was used. For unannotated PDB Ids, the Uniprot Id taken from the PDB file or the sequence information of each chain was used to find the appropriate gene ontology terms. The gene ontology terms were classified into nine groups based on each term's position on the gene ontology graph. Statistical enrichment of each group in the ID set relative to non-redundant PDB set was calculated based on hypergeometric distributions [70].

### Interface analysis

The interface of a protein complex was defined as the region with a change in solvent accessible surface area (SASA) upon binding. Similarly, we defined all residues with a change in SASA upon binding to be interface residues. For ID complexes and 3D complexes, the SASA was calculated for the PDB structure of the whole complex and the binding partners in isolation. The change in SASA upon protein binding is defined as:
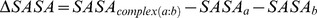
SASA was calculated with Areaimol [71], [72] using a probe radius of 1.4 Å. In addition to standard van der Waals radii used by Areaimol, the radii of zinc, calcium, and sodium ions were taken from CHARMM22 parameters [73]. Importantly, when calculating the size of the interfaces, we take the average between the two sides of the protein complex.

Interface residues were further divided into core and rim based on definitions by Levy [10]. The relative SASA (rSASA) was calculated by normalizing the residues' SASA by their SASA in a Gly-X-Gly peptide. Residues with rSASA greater than 0.25 in the complexed state were assigned to the rim. Residues that have rSASA less than 0.25 in the complexed state were assigned to the core if rSASA is greater than 0.25 in the uncomplexed state.

### High-resolution dataset

The high-resolution datasets of both 3D complex dimers and ID complexes consist of X-ray structures that are higher in resolution than 2.5 Å. We also excluded structures that have heterogens because they cannot be readily modeled using CHARMM and other programs. We included some of the ID complex structures containing heterogens with resolutions higher than 2.0 Å to maximize the size of the high-resolution ID set. Coordinate ions such as calcium and zinc were included in CHARMM structures. Other small molecules that are not part of the native structure or do not interact with the ID complex interface were ignored (e.g. glycerol from crystallization process). The high-resolution datasets are used for all the procedures described below.

### Salt bridge and hydrogen bond analysis

We defined salt bridges as any donor nitrogen and acceptor oxygen side chain atom pairs that are closer than 4.0 Å apart. Terminal charged groups were also included in the analysis. But despite the prevalence of terminal groups on relatively short ID segments, the terminal groups did not contribute significantly. Distances between each charged interface residue defined through SASA calculations and all other charged residues were calculated.

We used the program HBPlus to calculate hydrogen bonds from the high-resolution protein complex structures [74]. We used the default criteria and all hydrogen bonds formed across protein complex interfaces were tabulated. The analysis did not include aromatic hydrogen bonds.

### Alanine scan

FoldX version 3.0 beta3 was used to for the ALA-scan. Calculations were carried at 298K, pH 7, and ionic strength of 0.05M [75]. The FoldX repair procedure was used on each complex before introducing alanine mutations. ALA-scan using FoldX outputs a change in free energy of folding (ΔG_mut_) for each residue. The change in binding energy upon mutation (ΔΔG_bind_) for each of the interface residue was calculated by subtracting the ΔG_mut_ of the isolated protein subunit from that of the complex. Interface residues with a ΔΔG_bind_>1.5 kcal/mol were classified as interaction hot spots. Alanine, glycine, and proline residues were not analyzed.

### Electrostatic calculations

Polar solvation free energy and total electrostatic free energy of binding were calculated using DelPhi with a procedure similar to that of Sheinerman and Honig [20]. As discussed in their article, this is a simplified approximation that assumes the components of the complex do not undergo significant conformational changes upon binding. They described the free energy of binding (ΔG_bind_) as a sum of the free energy due to changes in conformation during binding (ΔG_strain_) and free energy of rigid binding (ΔG_rigid_). ΔG_strain_ consists of enthalpic and entropic changes upon binding and is always positive in value, which is unfavorable for binding [6]. Similar to their study, the electrostatic calculations performed in this study also consisted only of ΔG_rigid_.

We used the CHARMM param22 charges and radii for the calculations [73]. The scale is set to 2 grids/Å with a grid size of 401. The interior-dielectric and exterior-dielectric constants are 2 and 80, respectively. In accordance to the method used by Sheinerman and Honig, we used an interior dielectric constant of 2. We also tested the calculation with an interior dielectric constant of 4. We saw the same pattern in the free energies of binding among the ID complexes and the 3D complexes, but the magnitudes of the binding free energies were much smaller (not shown). For each complex, electrostatic calculations were done for the complex and both binding partners in isolation. All three structures were placed in the same grid, which was centered on the geometrical center of the protein complex. The probe radius for determining the solvent accessible surface area was 1.4 Å. Each calculation consisted of 2000 iterations of the linear Poisson-Boltzmann Equation (PBE). Generally, 1000 iterations were more than enough for our systems to converge.

Similar to the alanine scanning calculations, the electrostatic free energy of binding was calculated as the change in electrostatic free energy between the complex and the two subunits in the unbound state. Wang and Kollman [76] showed that the electrostatic contribution to the free energy of binding of proteins in water with no salt could be calculated as follows:
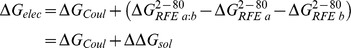
where 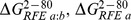, and 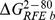 are the corrected reaction field energies of the whole complex (a:b), protein *a* in isolation, and protein *b* in isolation calculated by DelPhi with interior and exterior dielectric constants of 2 and 80, respectively. The subtraction of the reaction field energy of the protein subunits from the complex results in the change in free energy of solvation from binding. 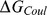 is the Coulombic interaction energy, which is calculated as:
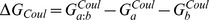
where 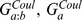 and 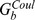 are the Coulombic energy of the complex, subunit *a*, and subunit *b* respectively.

The Coulombic and solvation energies were calculated in zero salt concentration. The ionic contributions were calculated by subtracting the total grid energy calculated in the 0.1M salt condition by the total grid energy in the zero-salt condition. In the 0.1M salt condition, an ion exclusion (Stern) layer of 2.0 Å surrounds the protein where the ion concentration is zero. In salt solution, the electrostatic contributions to the free energy of binding is equal to:
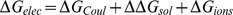
where 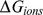 is the salt contribution to the electrostatic component of the binding free energy.

### Molecular modelling and structure minimization

The continuum electrostatic calculations were done on structures minimized in the bound state with CHARMM [77]. We used Param22 with topology and parameter files from the CHARMM-GUI [73], [78] and the FACTS implicit solvation model [79]. First, missing atoms were added with the build command. Subsequently, the structures were minimized. All backbone atoms were fixed at their crystal coordinates before applying 30 steps of steepest descent minimization at 0.05 kcal/mol gradient tolerance. Afterwards, all heavy atoms with coordinates in the original PDB file were constrained with force of 50 kcal/mol/Å^2^ before the whole structure was minimized with 5000 conjugate steps at 0.002 kcal/mol gradient tolerance.

## Supporting Information

Figure S1 Classifier performance analysis. (a) ROC curve for the Rg/N classifier. The true positive set contains complexes where one interaction partner is experimentally proven to be ID (ID complexes), and the negative set contains complexes of globular proteins from the 3D complex database (3D complexes). (b) ROC curve with disulfide-rich domains and coiled coils removed from the negative set (3D complexes) and the corresponding (c) Matthew's correlation coefficient (MCC) curve as a function of different Rg/N cutoffs.
(TIF)

Figure S2 Natural log of the radius of gyration (R_g_) plotted against natural log of protein length (N). 52 ID segments from the literature and 3D complex proteins are represented by red squares and black triangles, respectively. The slopes of the linear fits (red and black lines, respectively) provide the scaling factors ν of 0.5 and 0.35 for ID segments and 3D complex proteins, respectively.
(TIF)

Figure S3 Box plot of the distribution of the fraction of intrinsic disorder predicted from sequence of the selected protein segments. Shown here are the distribution of the fraction of predicted disorder for the selected ID segments and the selected ID segments extended by 30 amino acids on each side, respectively. The asterisk indicates that the distributions are significantly different (*p* values<0.05; Wilcoxon test).
(TIF)

Figure S4 Residue composition of the identified ID segments (red), the 52 ID segments from our literature search that we used to evaluate the classifier (green) as well as 1150 ID protein segments taken from the DisProt database (purple) relative to 3D complex protein.
(TIF)

Figure S5 Gene ontology analysis. (a) *P* values for the enrichment of gene ontology annotations among the proteins harboring the selected ID segments when compared to the annotations of the proteins in the non-redundant PDB dataset. (b) Gene ontology distribution of proteins harboring the selected ID segments.
(TIF)

Figure S6 Average number of each amino acid per complex partner in the core (a) and rim (b). The red, magenta, and grey columns represent ID segments, ID segment partner, and 3D complex protein interfaces, respectively.
(TIF)

Figure S7 (a) Scatter plots of change in solvent accessible surface area (SASA) against ΔΔG_bind_. The top, middle and bottom graphs show data points of hydrophobic, charged and salt-bridging residues of the BP in grey, red and blue circles, respectively. (b) and (c) are box plots of ΔΔG_bind_ of hydrophobic and charged interface residues, respectively, normalized by the change in solvent accessible surface area. ID segment residues are in red, BP residues are in purple, and 3D complex residues are in grey. Asterisks identify distributions that are significantly different (*p* values<0.05; Wilcoxon test).
(TIF)

Figure S8 Electrostatic components of the binding free energy. (a) Electrostatic contribution to the desolvation free energy of binding. (b) Coulombic interaction energy of binding. (c) Total electrostatic free energy of binding. Electrostatic contributions for 27 ID complexes found in the literature with high-resolution structures are in red and 109 3D complexes are in grey. Asterisks identify distributions that are significantly different (*p* values<0.05; Wilcoxon test).
(TIF)

Figure S9 Interface-normalized electrostatic components of the binding free energy. (a) Electrostatic contribution to the desolvation free energy of binding. (b) Coulombic interaction energy of binding. (c) Total electrostatic free energy of binding. Asterisks identify distributions that are significantly different (*p* values<0.05; Wilcoxon test).
(TIF)

Table S1 Performance of classifiers using varying scaling factors ν on the 52 ID complexes (positive set) and the 3D complex dataset (negative set). ^a^ Area under ROC curve. ^b^ Matthew's correlation coefficient. ^c^ False discovery rate. ^d^ Sensitivity.
(TIF)

Table S2 Performance of classifiers using varying scaling factors ν on the 52 ID complexes (positive set) and the 3D complex dataset without coiled coils and disulfide-rich domains (negative set).
(TIF)

Table S3 The number of residues in the core and rim regions of proteins in our datasets.
(TIF)
