# Supplementary figures and images for: On the Importance of Polar Interactions for Complexes Containing Intrinsically Disordered Proteins
Source: PLoS Comput Biol. 2013 Aug 22;9(8):e1003192. doi: 10.1371/journal.pcbi.1003192 (PMC3749945; doi:10.1371/journal.pcbi.1003192)

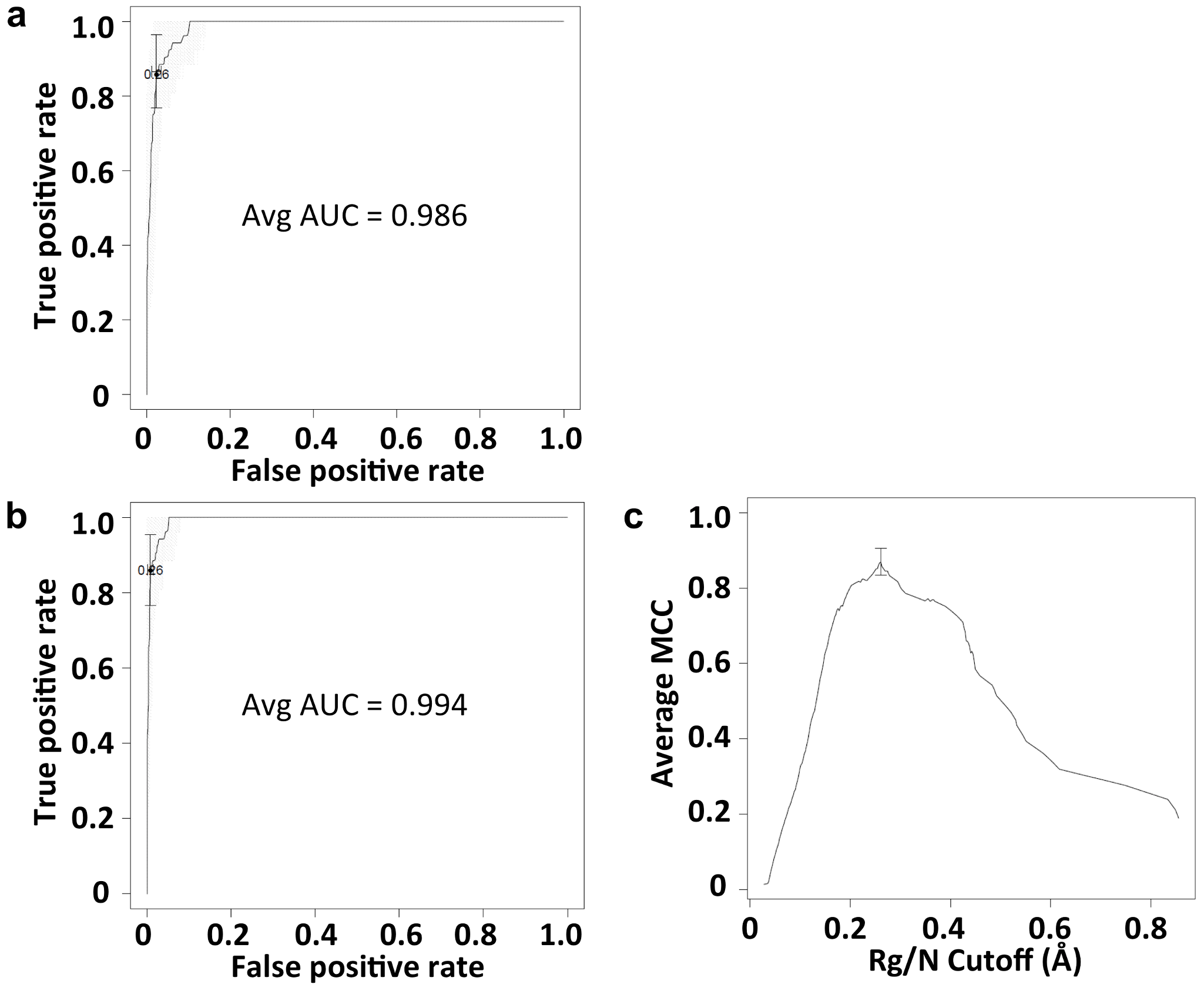

Supplement: Figure S1 — Classifier performance analysis. (a) ROC curve for the Rg/N classifier. The true positive set contains complexes where one interaction partner is experimentally proven to be ID (ID complexes), and the negative set contains complexes of globular proteins from the 3D complex database (3D complexes). (b) ROC curve with disulfide-rich domains and coiled coils removed from the negative set (3D complexes) and the corresponding (c) Matthew's correlation coefficient (MCC) curve as a function of different Rg/N cutoffs. (TIF) [file pcbi.1003192.s001.tif]

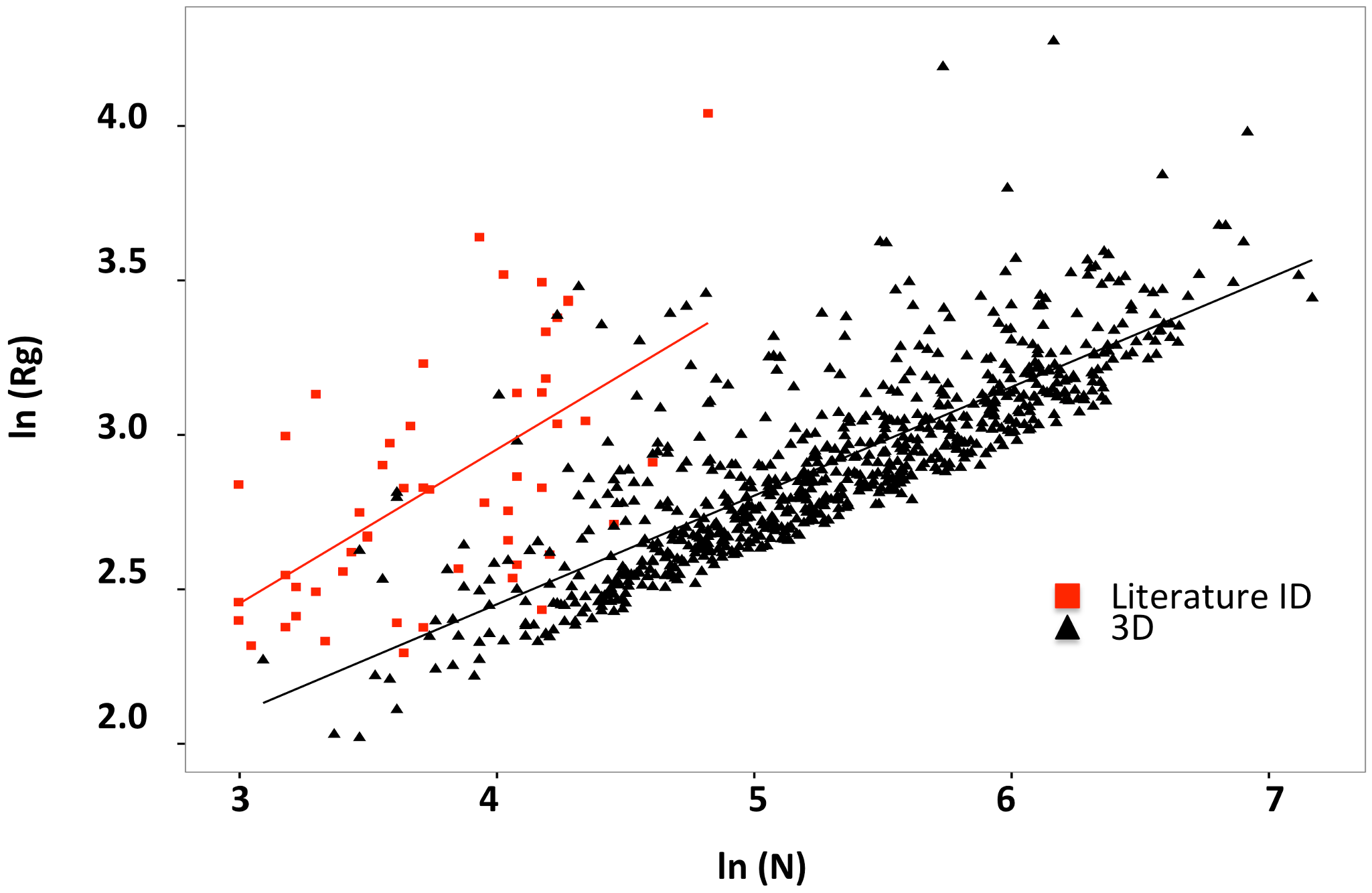

Supplement: Figure S2 — Natural log of the radius of gyration (Rg) plotted against natural log of protein length (N). 52 ID segments from the literature and 3D complex proteins are represented by red squares and black triangles, respectively. The slopes of the linear fits (red and black lines, respectively) provide the scaling factors ν of 0.5 and 0.35 for ID segments and 3D complex proteins, respectively. (TIF) [file pcbi.1003192.s002.tif]

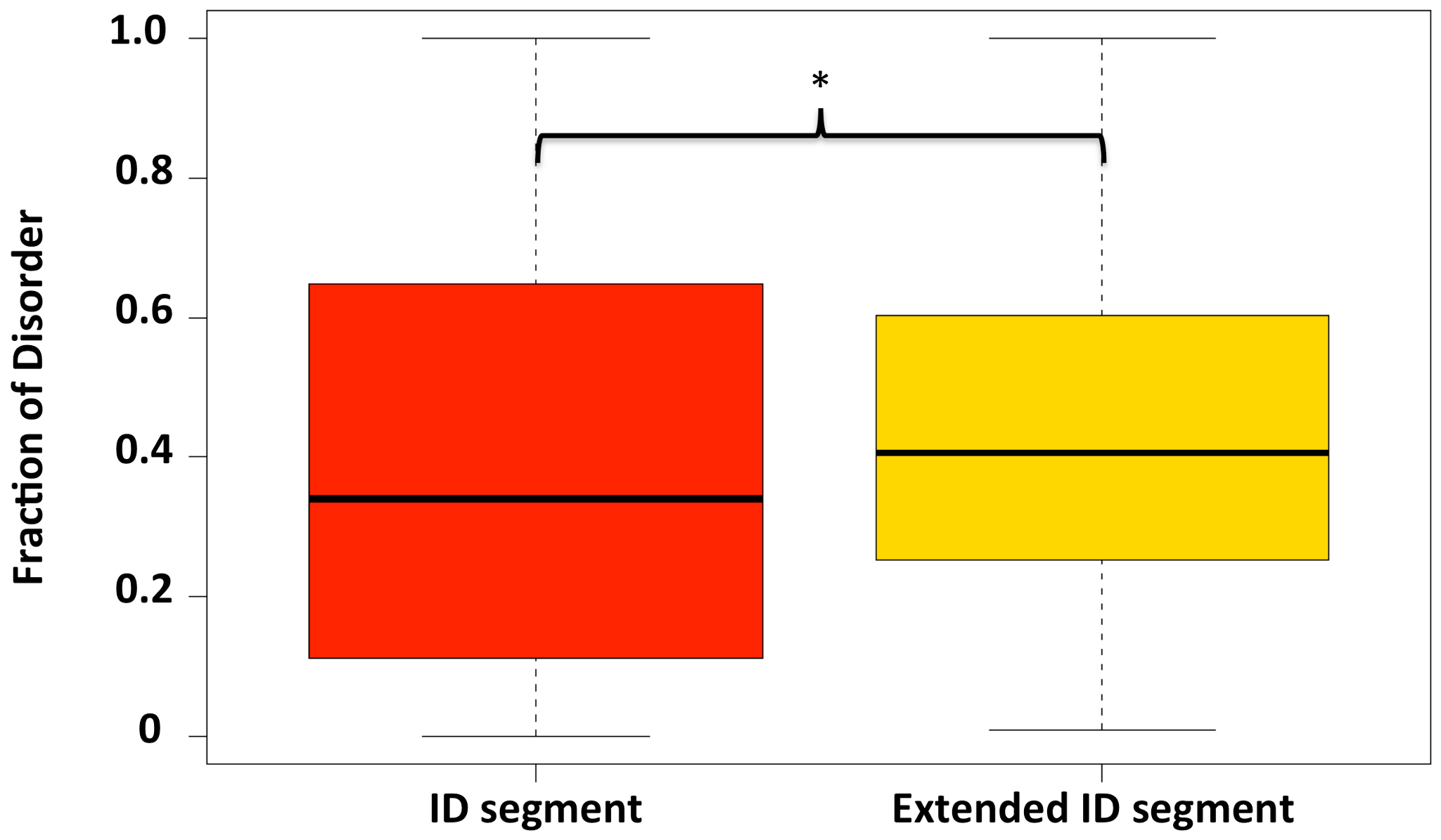

Supplement: Figure S3 — Box plot of the distribution of the fraction of intrinsic disorder predicted from sequence of the selected protein segments. Shown here are the distribution of the fraction of predicted disorder for the selected ID segments and the selected ID segments extended by 30 amino acids on each side, respectively. The asterisk indicates that the distributions are significantly different (p values<0.05; Wilcoxon test). (TIF) [file pcbi.1003192.s003.tif]

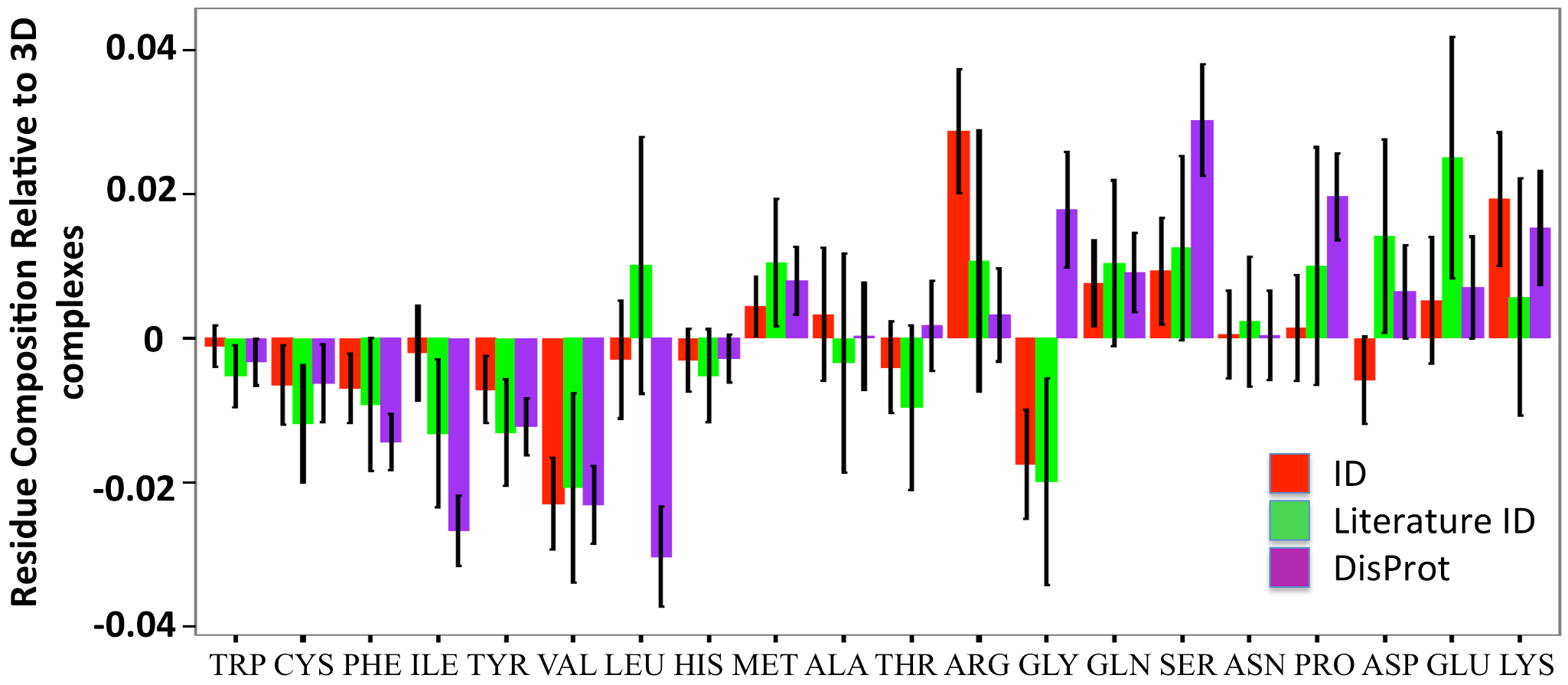

Supplement: Figure S4 — Residue composition of the identified ID segments (red), the 52 ID segments from our literature search that we used to evaluate the classifier (green) as well as 1150 ID protein segments taken from the DisProt database (purple) relative to 3D complex protein. (TIF) [file pcbi.1003192.s004.tif]

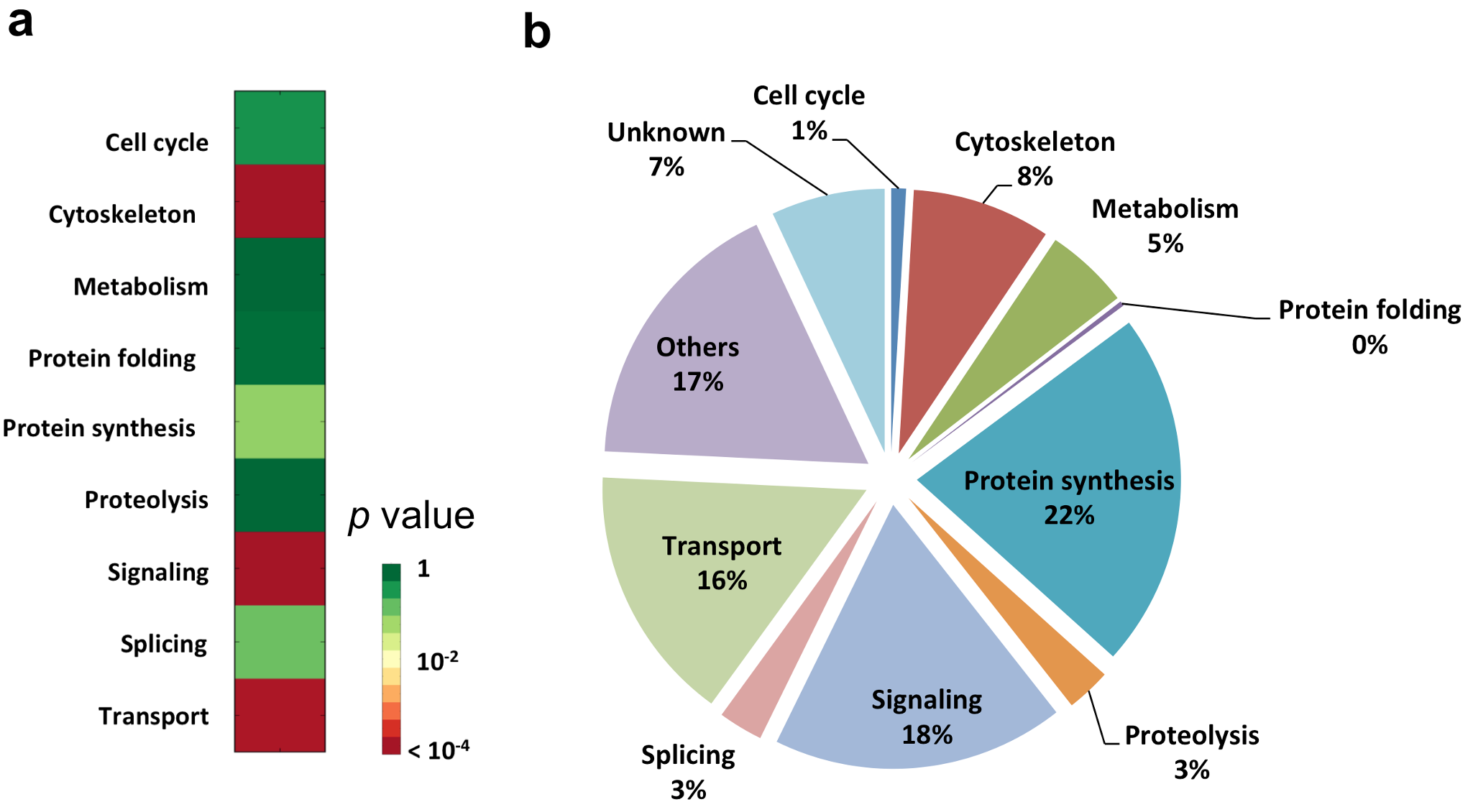

Supplement: Figure S5 — Gene ontology analysis. (a) P values for the enrichment of gene ontology annotations among the proteins harboring the selected ID segments when compared to the annotations of the proteins in the non-redundant PDB dataset. (b) Gene ontology distribution of proteins harboring the selected ID segments. (TIF) [file pcbi.1003192.s005.tif]

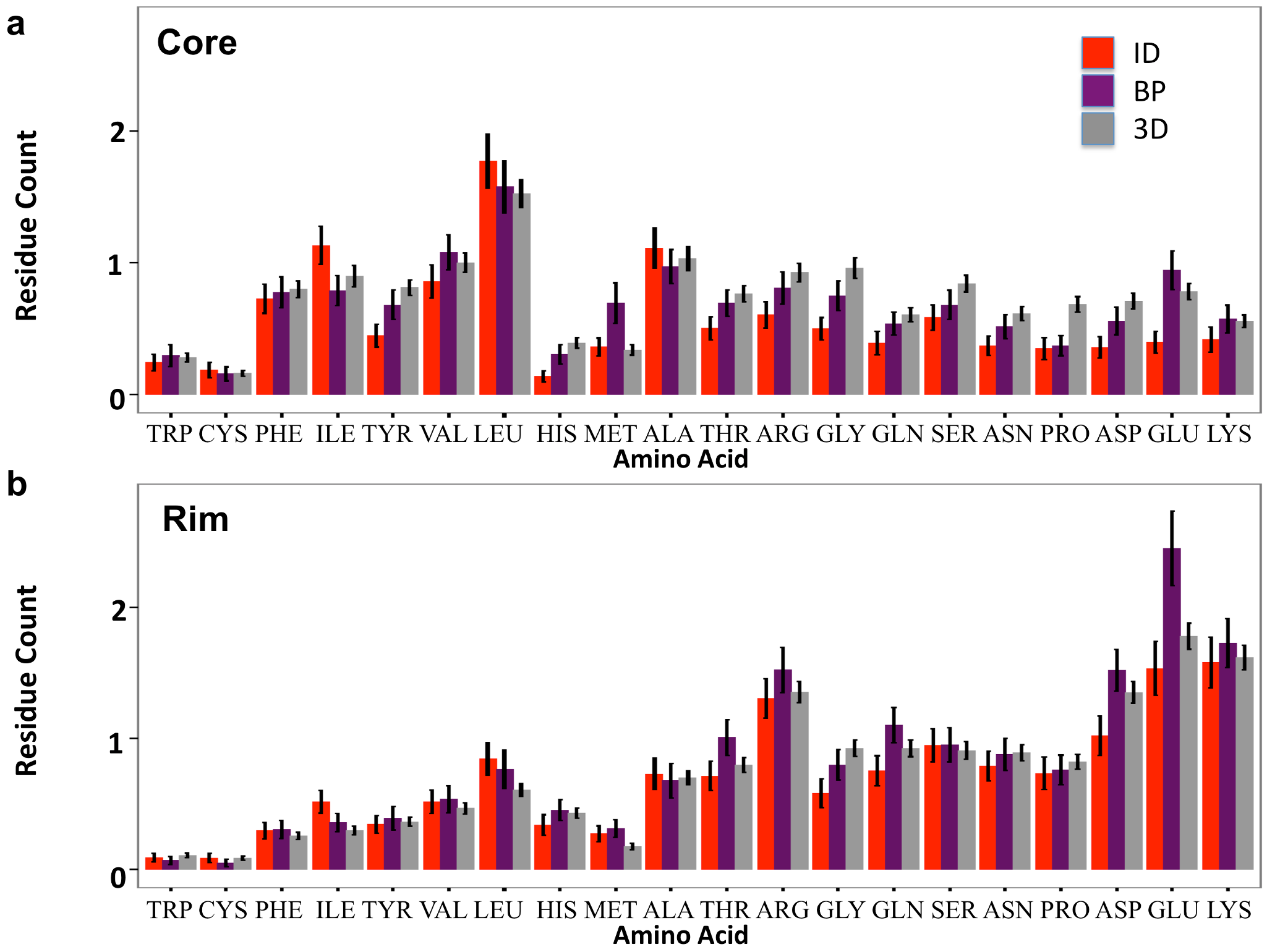

Supplement: Figure S6 — Average number of each amino acid per complex partner in the core (a) and rim (b). The red, magenta, and grey columns represent ID segments, ID segment partner, and 3D complex protein interfaces, respectively. (TIF) [file pcbi.1003192.s006.tif]

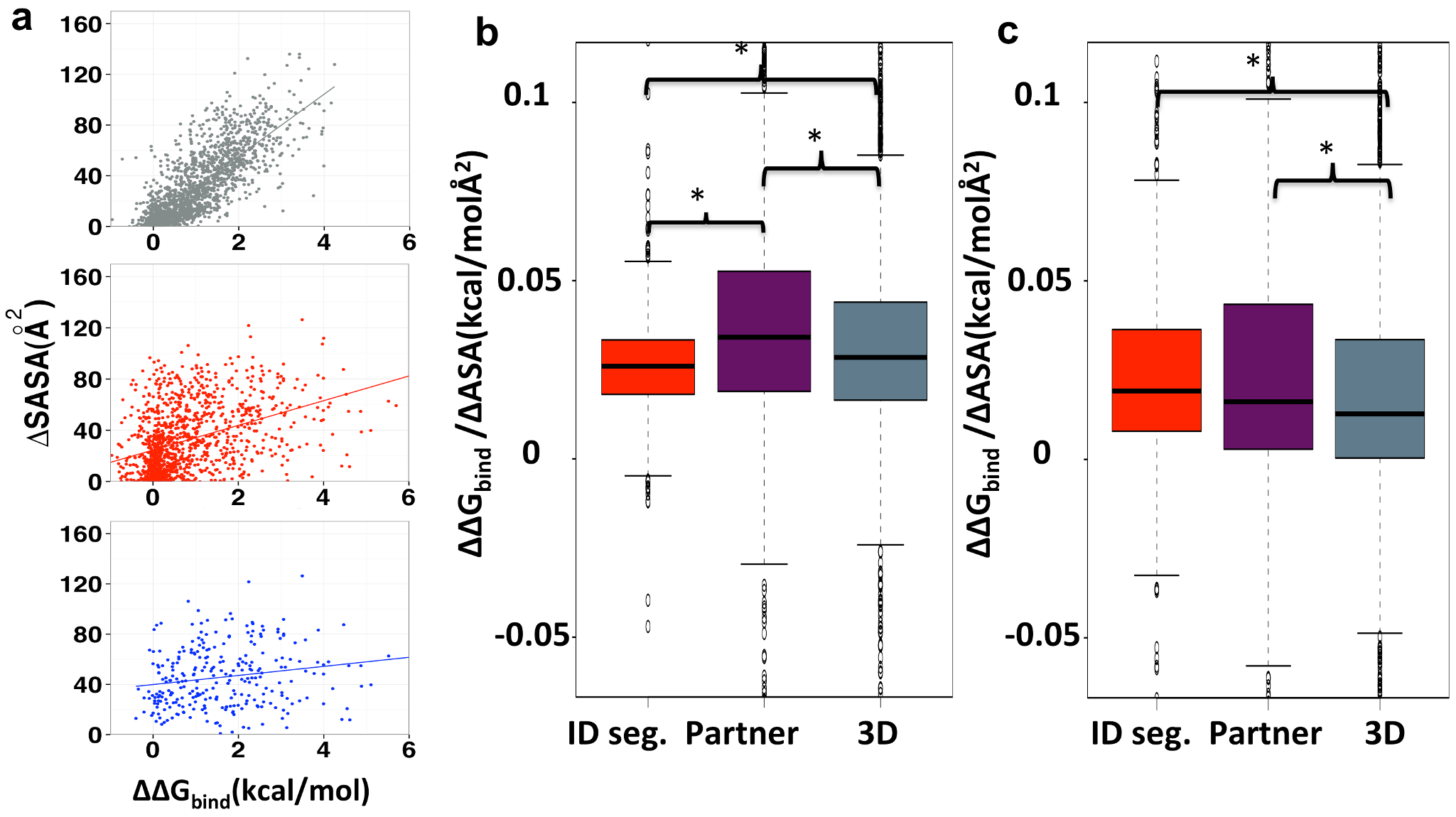

Supplement: Figure S7 — (a) Scatter plots of change in solvent accessible surface area (SASA) against ΔΔGbind. The top, middle and bottom graphs show data points of hydrophobic, charged and salt-bridging residues of the BP in grey, red and blue circles, respectively. (b) and (c) are box plots of ΔΔGbind of hydrophobic and charged interface residues, respectively, normalized by the change in solvent accessible surface area. ID segment residues are in red, BP residues are in purple, and 3D complex residues are in grey. Asterisks identify distributions that are significantly different (p values<0.05; Wilcoxon test). (TIF) [file pcbi.1003192.s007.tif]

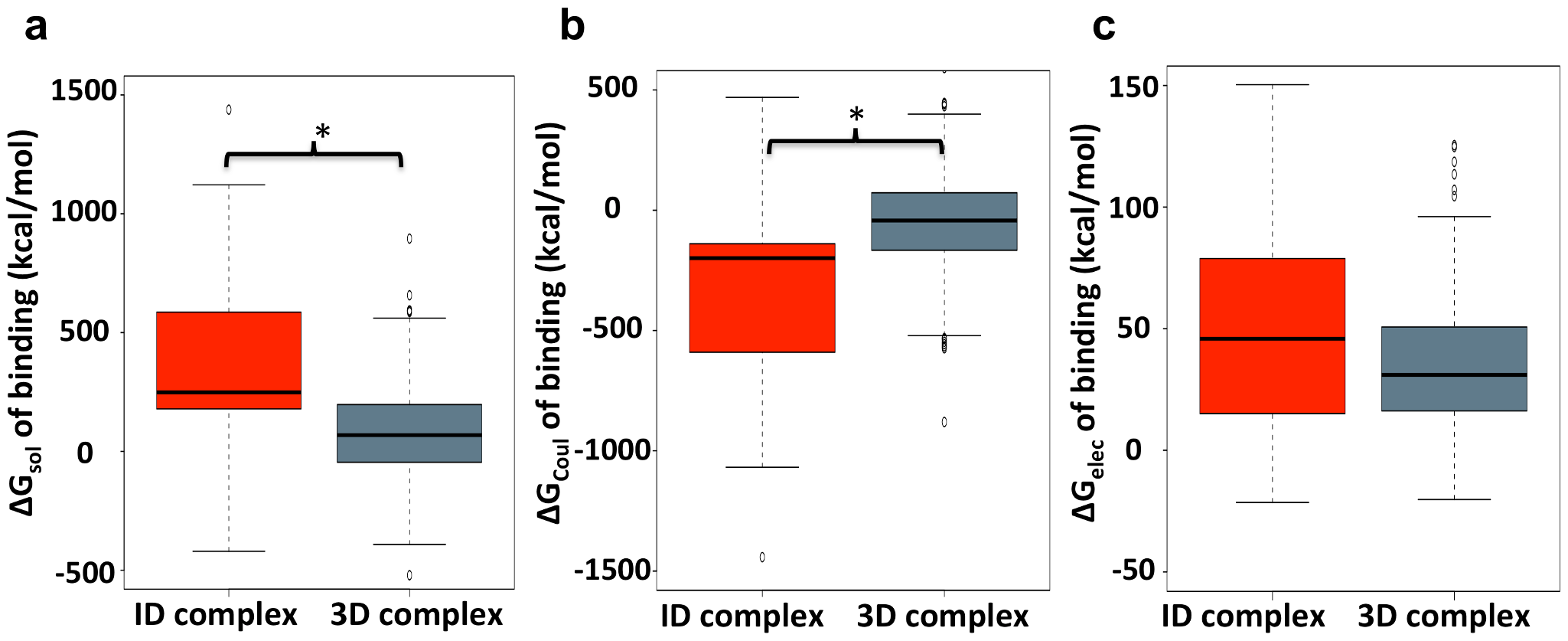

Supplement: Figure S8 — Electrostatic components of the binding free energy. (a) Electrostatic contribution to the desolvation free energy of binding. (b) Coulombic interaction energy of binding. (c) Total electrostatic free energy of binding. Electrostatic contributions for 27 ID complexes found in the literature with high-resolution structures are in red and 109 3D complexes are in grey. Asterisks identify distributions that are significantly different (p values<0.05; Wilcoxon test). (TIF) [file pcbi.1003192.s008.tif]

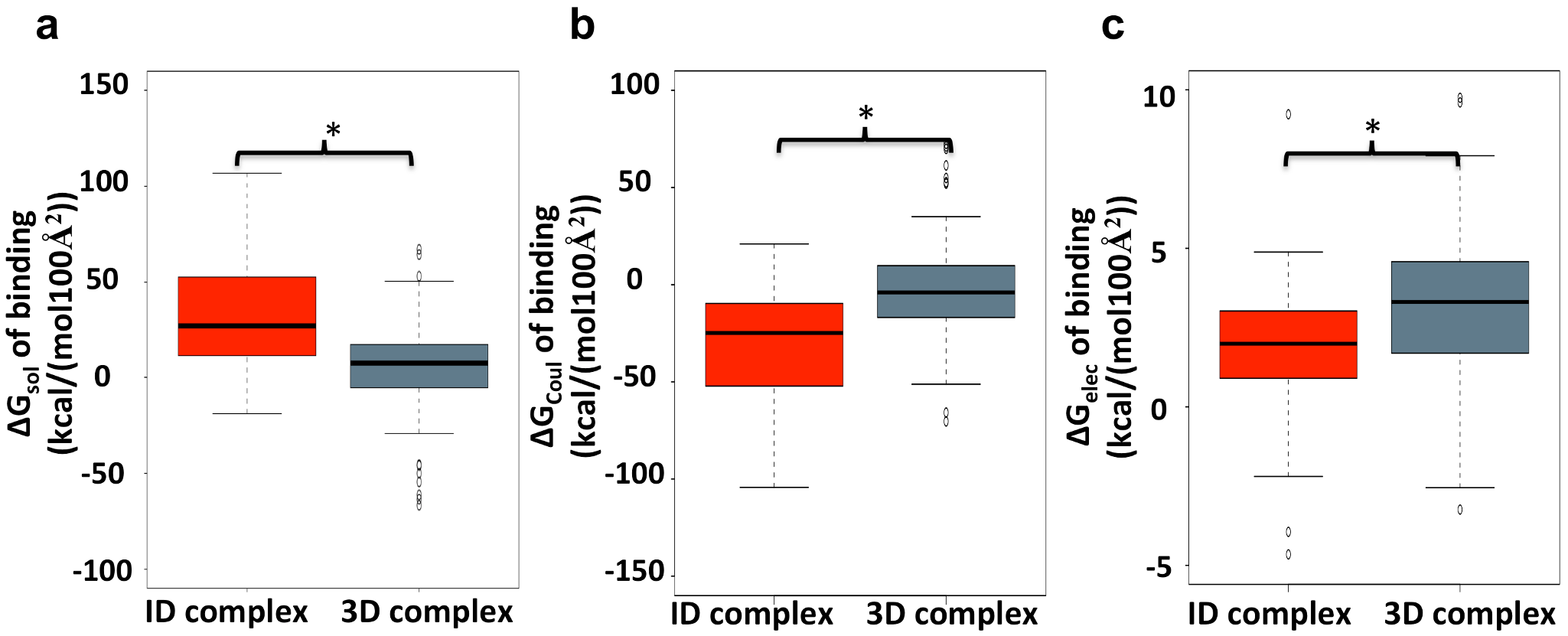

Supplement: Figure S9 — Interface-normalized electrostatic components of the binding free energy. (a) Electrostatic contribution to the desolvation free energy of binding. (b) Coulombic interaction energy of binding. (c) Total electrostatic free energy of binding. Asterisks identify distributions that are significantly different (p values<0.05; Wilcoxon test). (TIF) [file pcbi.1003192.s009.tif]

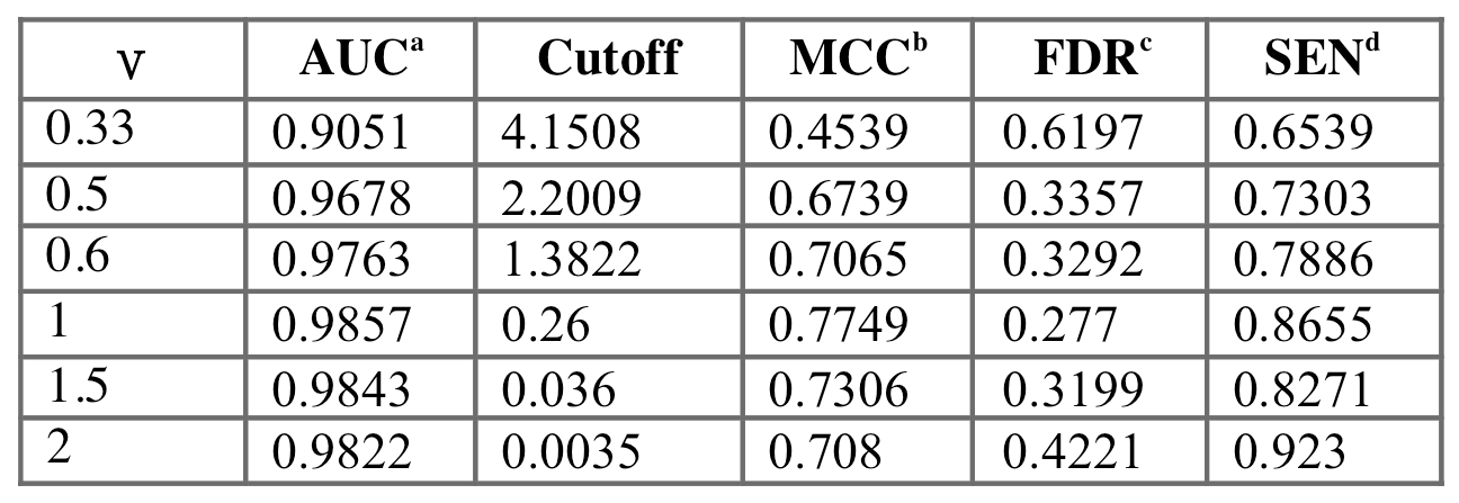

Supplement: Table S1 — Performance of classifiers using varying scaling factors ν on the 52 ID complexes (positive set) and the 3D complex dataset (negative set). a Area under ROC curve. b Matthew's correlation coefficient. c False discovery rate. d Sensitivity. (TIF) [file pcbi.1003192.s010.tif]

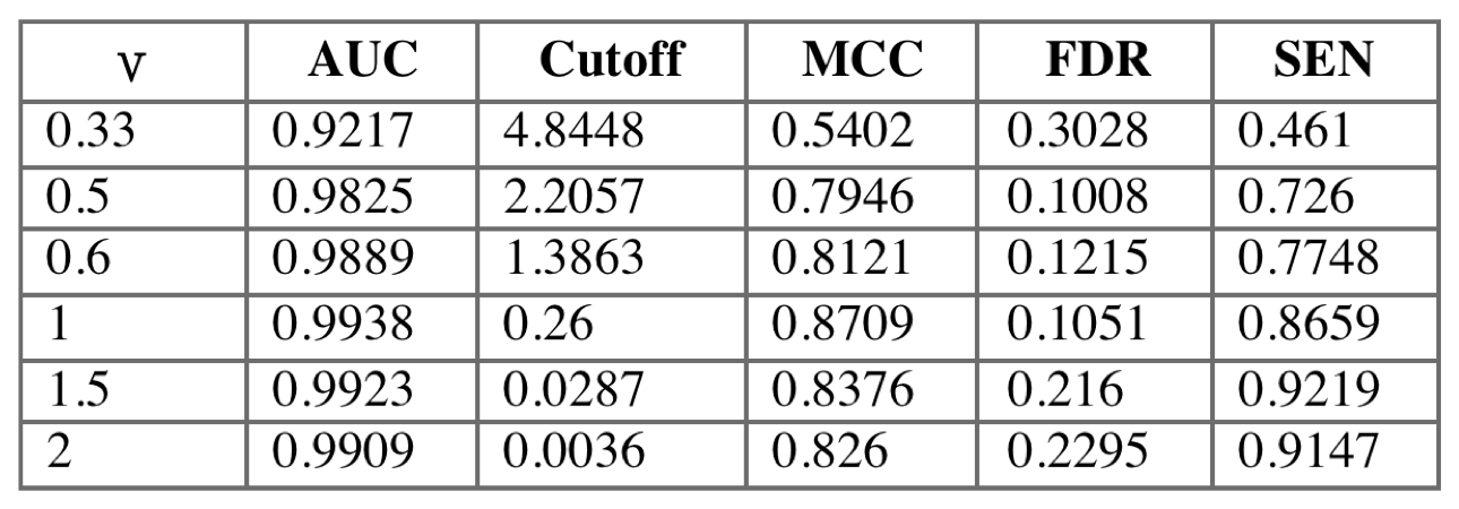

Supplement: Table S2 — Performance of classifiers using varying scaling factors ν on the 52 ID complexes (positive set) and the 3D complex dataset without coiled coils and disulfide-rich domains (negative set). (TIF) [file pcbi.1003192.s011.tif]

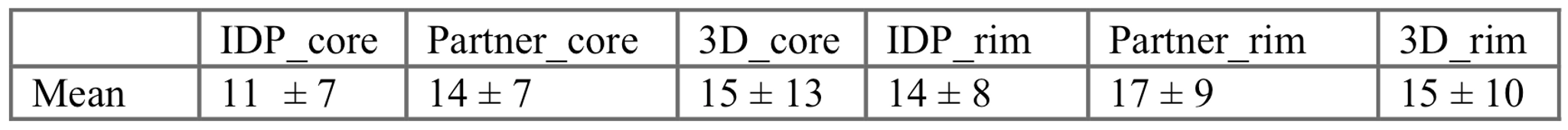

Supplement: Table S3 — The number of residues in the core and rim regions of proteins in our datasets. (TIF) [file pcbi.1003192.s012.tif]
